# Increased HIV-1 transcriptional activity and infectious burden in peripheral blood and gut-associated CD4^+^ T cells expressing CD30

**DOI:** 10.1371/journal.ppat.1006856

**Published:** 2018-02-22

**Authors:** Louise E. Hogan, Joshua Vasquez, Kristen S. Hobbs, Emily Hanhauser, Brandon Aguilar-Rodriguez, Rajaa Hussien, Cassandra Thanh, Erica A. Gibson, Alexander B. Carvidi, Louis C. B. Smith, Shahzada Khan, Martin Trapecar, Shomyseh Sanjabi, Ma Somsouk, Cheryl A. Stoddart, Daniel R. Kuritzkes, Steven G. Deeks, Timothy J. Henrich

**Affiliations:** 1 Division of Experimental Medicine, University of California San Francisco, San Francisco, California, United States of America; 2 Virology and Immunology, Gladstone Institutes, San Francisco, California, United States of America; 3 Department of Microbiology and Immunology, University of California San Francisco, San Francisco, California, United States of America; 4 Division of Gastroenterology, Department of Medicine, University of California San Francisco, San Francisco, California, United States of America; 5 Division of Infectious Diseases, Brigham and Women's Hospital, Harvard Medical School, Boston, Massachusetts, United States of America; 6 Positive Health Program, Department of Medicine, University of California San Francisco, San Francisco, California, United States of America; Vaccine Research Center, UNITED STATES

## Abstract

HIV-1-infected cells persist indefinitely despite the use of combination antiretroviral therapy (ART), and novel therapeutic strategies to target and purge residual infected cells in individuals on ART are urgently needed. Here, we demonstrate that CD4^+^ T cell-associated HIV-1 RNA is often highly enriched in cells expressing CD30, and that cells expressing this marker considerably contribute to the total pool of transcriptionally active CD4^+^ lymphocytes in individuals on suppressive ART. Using *in situ* RNA hybridization studies, we show co-localization of CD30 with HIV-1 transcriptional activity in gut-associated lymphoid tissues. We also demonstrate that *ex vivo* treatment with brentuximab vedotin, an antibody-drug conjugate (ADC) that targets CD30, significantly reduces the total amount of HIV-1 DNA in peripheral blood mononuclear cells obtained from infected, ART-suppressed individuals. Finally, we observed that an HIV-1-infected individual, who received repeated brentuximab vedotin infusions for lymphoma, had no detectable virus in peripheral blood mononuclear cells. Overall, CD30 may be a marker of residual, transcriptionally active HIV-1 infected cells in the setting of suppressive ART. Given that CD30 is only expressed on a small number of total mononuclear cells, it is a potential therapeutic target of persistent HIV-1 infection.

## Author summary

Previous studies have shown that higher levels of soluble CD30 are associated with HIV-1 disease progression. Many of these studies, however, were performed prior to the implementation of combination ART, and the relationship between surface CD30 expression, soluble CD30 and HIV-1 infection in ART suppressed individuals, or those with viremic control off ART, is not known. We demonstrate that cell-associated HIV-1 RNA is highly enriched in CD4^+^ T cells expressing CD30, a member of the tumor necrosis factor receptor superfamily. These findings were observed in several HIV-1 infected donor groups, regardless of whether or not the participants were receiving suppressive ART. Furthermore, we demonstrate that *ex vivo* treatment with brentuximab vedotin, an antibody-drug conjugate that targets CD30, reduces the total amount of HIV-1 DNA in PBMC obtained from infected individuals. Finally, we show through *in situ* RNA hybridization studies that CD30 and HIV transcriptional activity co-localize in cells from gut biopsies obtained from HIV-1 infected donors. These data suggest that CD30 may be a marker of residual, transcriptionally active HIV-1 infected cells in the setting of suppressive ART.

## Introduction

CD30, a member of the TNF receptor superfamily, is expressed on tumor cells found in Hodgkin and other aggressive lymphomas [1,2] but only on a very small percentage of lymphocytes in healthy individuals [2,3,4,5,6,7]. Although its functions are largely undefined, CD30 has been implicated in the activation, proliferation and death of selected cell populations [2,4,8,9]. Stimulation of this receptor has been shown to activate NF-κB, a protein complex that regulates immune responses [2,9,10]. Infections with viral pathogens, such as human T-cell lymphotropic virus (HTLV), Epstein-Barr virus (EBV), and poxviruses markedly increase surface expression of CD30 compared with cytokine activation alone [4,11,12]. Given the rarity of CD30 expression *in vivo*, the dramatic increases in the setting of these infections may be secondary to virus-specific cell stress responses.

Prior studies have demonstrated that stimulation of CD30 results in increased HIV-1 expression *ex vivo* in cells obtained from untreated individuals, and higher levels of soluble CD30 are associated with HIV-1 disease progression [4,5,8,13,14,15,16,17,18,19,20]. However, many of these studies were performed in the setting of untreated HIV-1 infection and the relationship between surface CD30 expression, soluble CD30 (sCD30) and viral persistence in ART suppressed individuals or those with viremic control off ART is unknown. It is possible that CD30 is expressed on transcriptionally active HIV-1-infected residual cells in the setting of ART. Therefore, we investigated the relationship between CD30 and HIV-1 burden in peripheral blood and gut-associated lymphoid tissue from HIV-1-infected individuals on and off therapy. Overall, we demonstrate that HIV-1 infected CD30^+^ T cells markedly contribute to the total population of HIV-1 infected and transcriptionally active CD4^+^ T cells in several individuals regardless of ART use. In addition, we observed that CD30 and HIV-1 transcriptional activity co-localized in gut-associated lymphoid tissues in individuals on or off ART. Finally, we showed that brentuximab vedotin, significantly reduced the total amount of HIV-1 DNA in PBMC obtained from infected individuals.

## Results

### CD30 expression on CD4^+^ T cells is increased in HIV-1 infection

**Fig 1:**
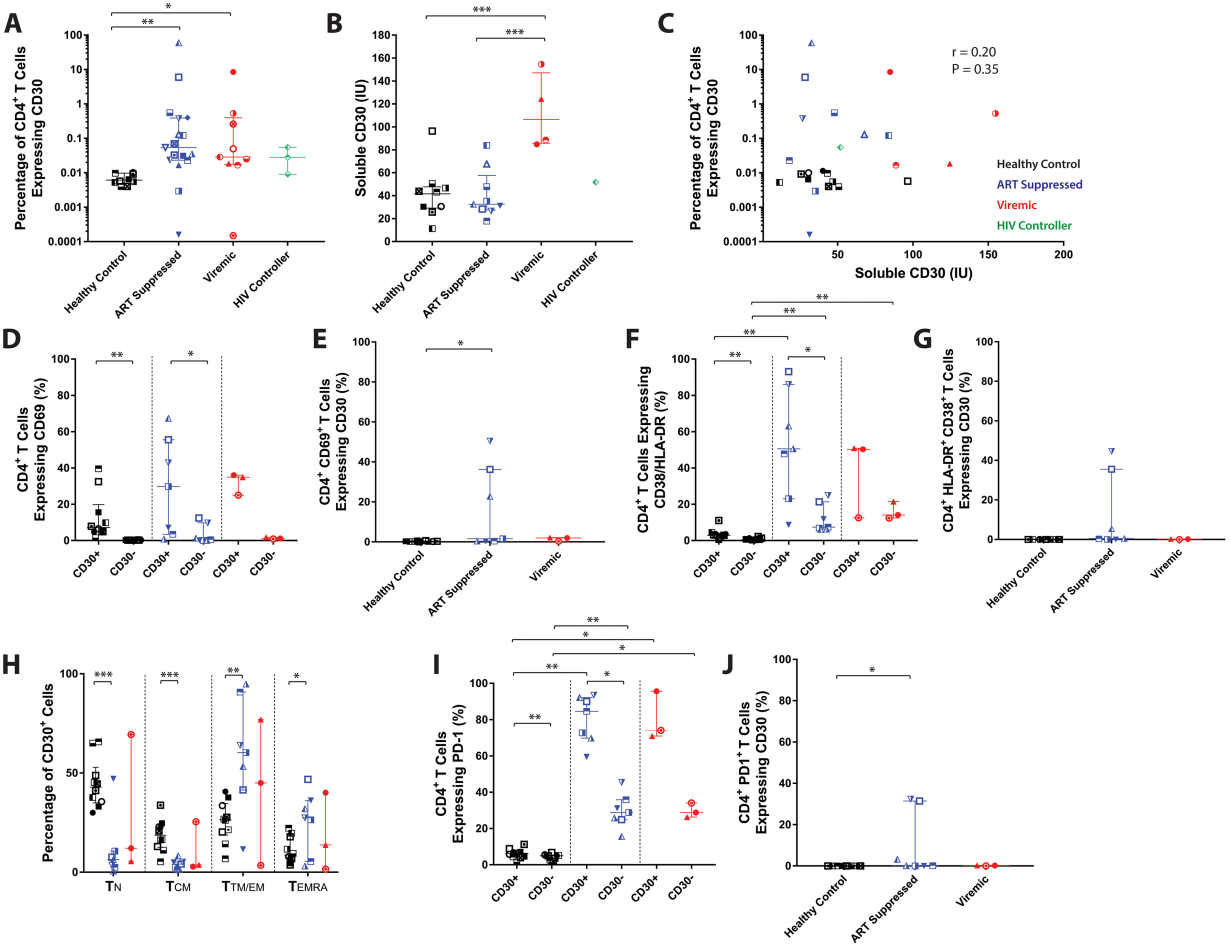
Assessment of CD30 expression on CD4^+^ T cells. (**A**) Percentage of CD4^+^ T cells expressing CD30 in samples from HIV-1-uninfected (n = 10) ART suppressed (n = 17) and viremic donors (n = 9) are shown. Surface expression was higher in ART suppressed and viremic HIV-1-infected individuals (p = 0.0023 and p = 0.045 respectively). **(B)** sCD30 (IU) in plasma was highest in viremic (n = 4) compared with HIV-1-uninfected (n = 10) and ART suppressed donors (n = 9) (p<0.001 and p<0.001 respectively). (**C**) No significant correlations were identified between the percentage of CD4^+^CD30^+^ T cells and sCD30 levels (r = 0.20, P = 0.35 by Spearman rank correlation analysis). The percentage of CD4^+^ T cells co-expressing CD69 in each cohort are shown in (**D**) and the percentage of CD69^+^CD4^+^ T cells expressing CD30 are shown in (**E**). The percentage of CD4^+^ T cells co-expressing HLA-DR (**F**) and HLA-DR^+^CD4^+^ T cells expressing CD30 (**G**) are shown. Despite significant increases in CD30^+^ cells expressing CD38/HLA-DR, there were no significant differences in the frequency of CD30 expression in CD38/HLA-DR-expressing CD4^+^ T cell populations. (**H**) The highest frequency of CD30^+^ T cells observed in HIV-1-infected individuals were of transitional/effector memory phenotype, compared to naïve T cells in healthy controls. (**I**) CD30^+^CD4^+^ and CD30^-^CD4^+^ T cells from viremic donors expressed significantly more PD1 than healthy controls (p = 0.02 and p = 0.02 respectively), CD30^+^CD4^+^ and CD30^-^CD4^+^ T cells from ART suppressed donors also expressed significantly more PD1 than healthy controls (p = 0.002 and p = 0.002 respectively). **(J)** Conversely, very few CD4^+^PD1^+^ T cells co-expressed CD30, but ART suppressed donors had significantly higher expression compared to healthy controls (p = 0.01). T Bars represent median ± interquartile range for all data. *p<0.05; **P < 0.01; ***P < 0.001. Significant intergroup differences were determined using rank Kurskal-Wallis tests incorporating Dunn's tests for multiple comparisons. Wilcoxon matched-pairs signed rank tests were used to determine statistical significant between CD30 intergroup, paired samples.

We analyzed the peripheral blood of HIV-1 infected and uninfected individuals (S1 Table, S1 Fig) for CD30 expression on PBMC, and plasma sCD30 levels. The frequency of CD30^+^CD4^+^ T cells was significantly higher among viremic and ART-suppressed HIV-1 infected groups, irrespective of ART regimen, compared to HIV-1-uninfected controls (p = 0.045 and p = 0.002 respectively). However, no significant differences in the frequency of CD30^+^CD4^+^ T cells were observed between the untreated and treated HIV-1 infected cohorts (Fig 1A). Plasma soluble CD30 levels were significantly higher in the viremic donor group as compared to the suppressed and uninfected group (p<0.001 and p<0.001 respectively) (Fig 1B), but sCD30 levels did not correlate significantly with CD30 surface expression (Fig 1C).

### CD30 expression is preferentially expressed on activated effector memory cells in HIV-1 infected individuals

The relationship between CD30 expression and immunological phenotypes in the setting of HIV-1 infection was determined on fresh peripheral blood CD4^+^ T cells isolated from HIV-1-infected and uninfected individuals. Both the frequencies of CD69 and co-expression of HLA-DR and CD38 were significantly increased in healthy controls and ART suppressed participants within CD30^+^CD4^+^ T cells compared to the CD30^-^CD4^+^ T cells (CD69; p = <0.002 and p = 0.001, respectively; HLA-DR; p = 0.002 and p = 0.03, respectively) (Fig 1D and 1F). The expression of CD30 is known to increase during many biological processes, including cellular activation [21]. However, the expression of CD30 is rare in individuals without concomitant viral infection or specific hematological malignancies. Therefore, to clarify whether we were simply selecting a highly activated population of CD4^+^ T cells, we determined the percentage of CD69^+^, HLA-DR^+^ or PD-1^+^ cells that expressed CD30 in addition to the percentages of CD30 that express these markers. Our data showed that few cells expressing markers associated with early or late activation (CD69 and CD38/HLA-DR, respectively) expressed CD30 (Fig 1E and 1G).

CD30 is thought to be up-regulated on activated CD4^+^ T cells, and while studies have demonstrated this following artificial stimulation *in vitro* [21,22,23], we are unaware of any data examining CD30 expression on unstimulated, non-malignant cells *ex vivo*. Therefore, we determined the expression of CD30 on unstimulated CD4^+^ T cell subsets in HIV-uninfected individuals. Interestingly, CD30 expression in HIV-uninfected donors was observed primarily on naïve CD4^+^ T cells, whereas most CD30-expressing CD4^+^ T cells in HIV-1-infected individuals, on or off ART, were found to be of effector/transitional memory phenotype (Fig 1H). A significantly higher percentage of CD30-expressing cells in healthy controls and HIV-1 infected donors also co-expressed PD-1 (p = 0.0020, p = 0.015), but again, less than 10% of all PD-1 expressing cells co-expressed CD30 (Fig 1I and 1J).

### HIV-1 enrichment in CD30-expressing CD4^+^ T cells

**Fig 2:**
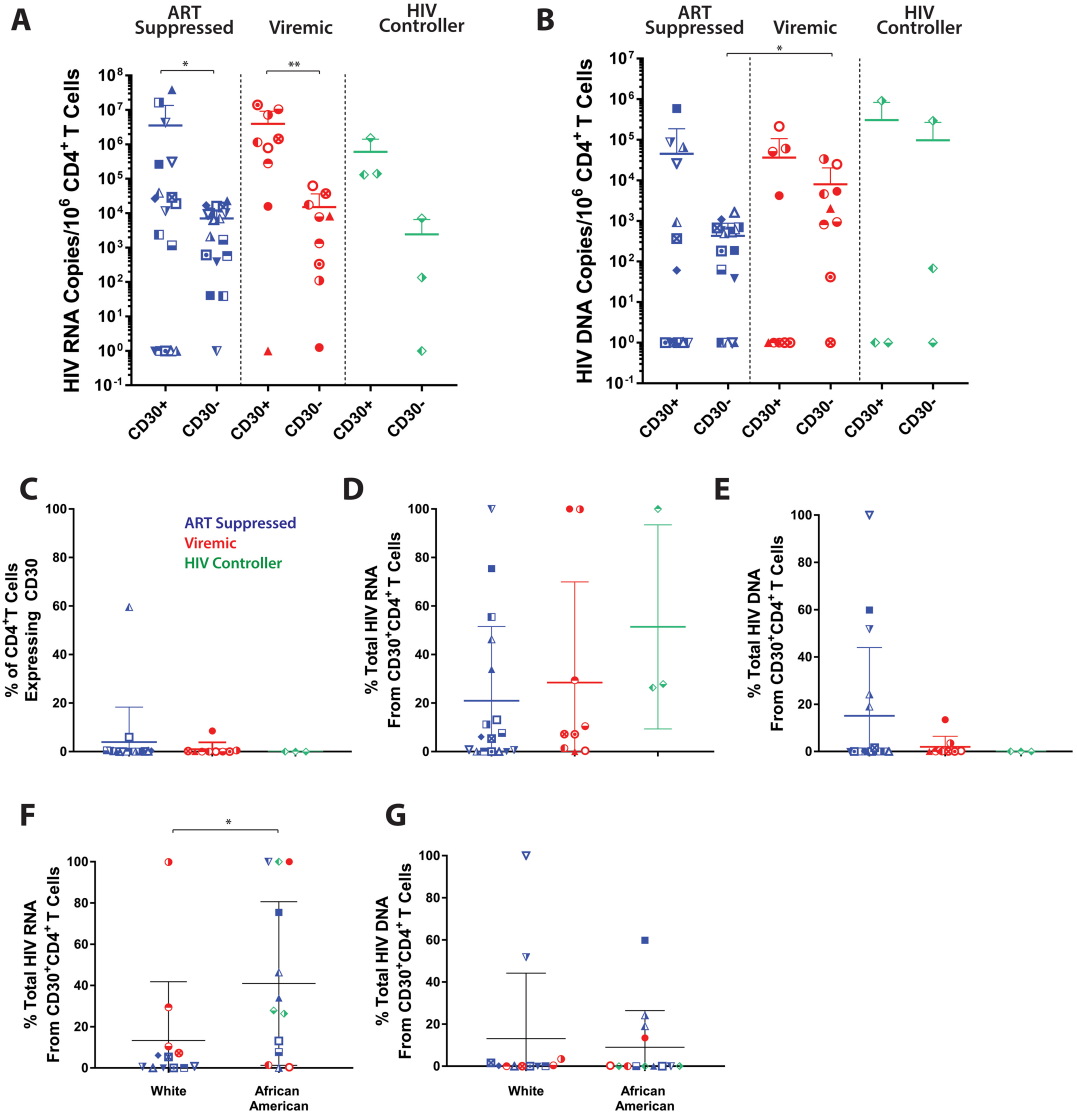
HIV-1 is enriched in CD30 expressing CD4^+^ T cells. Cell-associated HIV-1 unspliced RNA **(A)** and DNA **(B)** in sorted CD4^+^ T cell populations, in samples from ART suppressed (n = 17) and viremic donors (n = 9) are shown. HIV-1 RNA is significantly enriched in CD30 expressing cells from ART suppressed donors (p = 0.008) and viremic donors (p = 0.007). (C) The contribution of CD30^+^CD4^+^ T cells to total CD4^+^ T cell population, and the contribution of HIV-1 RNA **(D)** and DNA **(E)** from CD30^+^CD4^+^ sorted cells to total HIV-1 RNA and DNA burden in CD4^+^ T cells, are shown. Despite the rarity of CD30^+^ T cells, a large contribution of HIV-1 DNA and RNA are attributed to these cells in some individuals. The percentage of total HIV-1 RNA **(F)** but not DNA **(G)** found in CD30^+^CD4^+^ T cells was significantly higher in African American (n = 13) than white (n = 12) participants (p = 0.0103). Bars represent mean ± standard deviation; *P < 0.05; **P < 0.01; Significant intergroup differences were determined using Kruskal-Wallis tests incorporating Dunn's tests for multiple comparisons. Wilcoxon matched-pairs signed rank tests were used to determine statistical significant between CD30 intergroup, paired samples.

To assess the relationship between CD30 expression, HIV-1 transcriptional activity, and cell-associated HIV-1 DNA and RNA levels, we utilized fluorescence activated cell sorting to isolate CD30^+^CD4^+^ and CD30^-^CD4^+^ T cells obtained from 29 HIV-1-infected donors on suppressive ART (n = 17), viremic donors with high viral loads (n = 9), and individuals able to control HIV-1 without ART (HIV-1 controllers, n = 3; HIV-1 plasma RNA levels <500 copies/mL). HIV-1 genomic DNA and unspliced RNA were quantified by PCR for each subset [24,25]. Cell-associated HIV-1 RNA was significantly enriched within the CD30^+^CD4^+^ T cell population (p = 0.007 and p = 0.008 for ART suppressed and viremic groups, respectively) (Fig 2A and 2B). Despite CD30^+^ cells having several orders of magnitude higher HIV-1 DNA levels in samples from several individuals, no intragroup statistical significance was identified. Samples from all participants were included in the analysis, regardless of CD30 recovery from sorting (which at times was low, e.g. <50 cells) or if HIV-1 RNA or DNA were not detected in cells from these participants to reduce selection bias.

### CD30 and HIV-1 transcriptional activity

In order to determine the contribution of CD30^+^CD4^+^ T cells to the total RNA and DNA burden of HIV-1 infection in peripheral CD4^+^ T cell compartments, we compared the total HIV-1 RNA and DNA recovered from each T cell subset, taking into account the number of cells obtained, from ART suppressed (n = 17), viremic donors (n = 9) and HIV controllers (n = 3). The average percentage of CD4^+^ T cells expressing CD30 was found to be 3.97%, 1.05% and 0.03% in suppressed, viremic and controller groups, respectively (Fig 2C). Despite the rarity of these cells, an average of 21%, 28.5% and 51.4% HIV-1 RNA was attributed to CD30^+^CD4^+^ T cells in suppressed, viremic and controller groups, respectively. Strikingly, >90% of detectable cell-associated HIV-1 RNA was found within CD30^+^CD4^+^ T cells in samples from five individuals either on or off ART (Fig 2D). The largest contribution of CD30^+^ T cells to the peripheral HIV-1 DNA burden was observed in ART suppressed individuals, and 18.3%, 2.2% and 0.05% of HIV-1 DNA was attributed to CD30^+^CD4^+^ T cells in suppressed, viremic, and controller donor groups, respectively (Fig 2E). Moreover, in three ART-suppressed individuals, >50% of peripheral cell-associated HIV-1 DNA was found within CD30^+^CD4^+^ T cells. Overall, there was a high degree of inter-participant variability in the contribution of CD30^+^CD4^+^ T cells to the peripheral HIV-1 burden.

No significant relationship between the frequency of CD30^+^CD4^+^ T cells and CD4^+^ T cell count, age, or number of years since diagnosis was identified in non-parametric analyses (all P>0.05). However, the contribution of CD30^+^CD4^+^ T cells to the total pool of CD4^+^ T cell-associated HIV-1 RNA was found to be significantly higher in African American individuals (mean = 42.7%) compared to white individuals (mean 12.8%), irrespective of ART regimen (P = 0.0103) (Fig 2F). There were no significant differences in HIV-1 DNA contribution between racial groups (Fig 2G).

In order to verify that the rare population of CD30^+^ cells obtained from cell sorting was not from non-specific staining or other flow cytometric artefact, we performed mRNA gene expression PCR assays incorporating mRNA for CD3 complex genes, CD4, CD8α, and CD80 (a surface marker expressed on B cells and non-lymphocyte cells) on 5 additional sorted blood samples (S2 Fig). These additional extractions did not involve the use of HIV-uninfected carrier cells, in order to obtain undiluted human RNA from CD30^+^ and CD30^-^ cells. Overall, a majority of 2^-ΔΔCt^ values from the CD3 and CD4 mRNA PCR arrays comparing CD30^+^ to CD30^-^ CD4^+^ T cells were equal to or greater than one, suggesting that CD30^+^ cells have similar, if not higher levels of these mRNA transcripts. 2^-ΔΔCt^ is a measure of difference between two populations within individual samples based on the cycle threshold values obtained by quantitative PCR. A value of one indicates no difference and values greater than one indicate a greater number of RNA transcripts. In contrast, CD8 and CD80 mRNA were not detected in CD30^+^ cells from 3 and 4 samples, respectively (S2 Fig) whereas CD80 and CD8 mRNA could be detected in all of the sorted CD30^-^ cell populations. These results indicate that the sorted CD30^+^ cells have mRNA profiles consistent with CD3^+^CD4^+^ lymphocytes.

### Lymphocyte phenotypes and HIV-1 burden in rectal CD4^+^ T cells expressing CD30 and CD32

**Fig 3:**
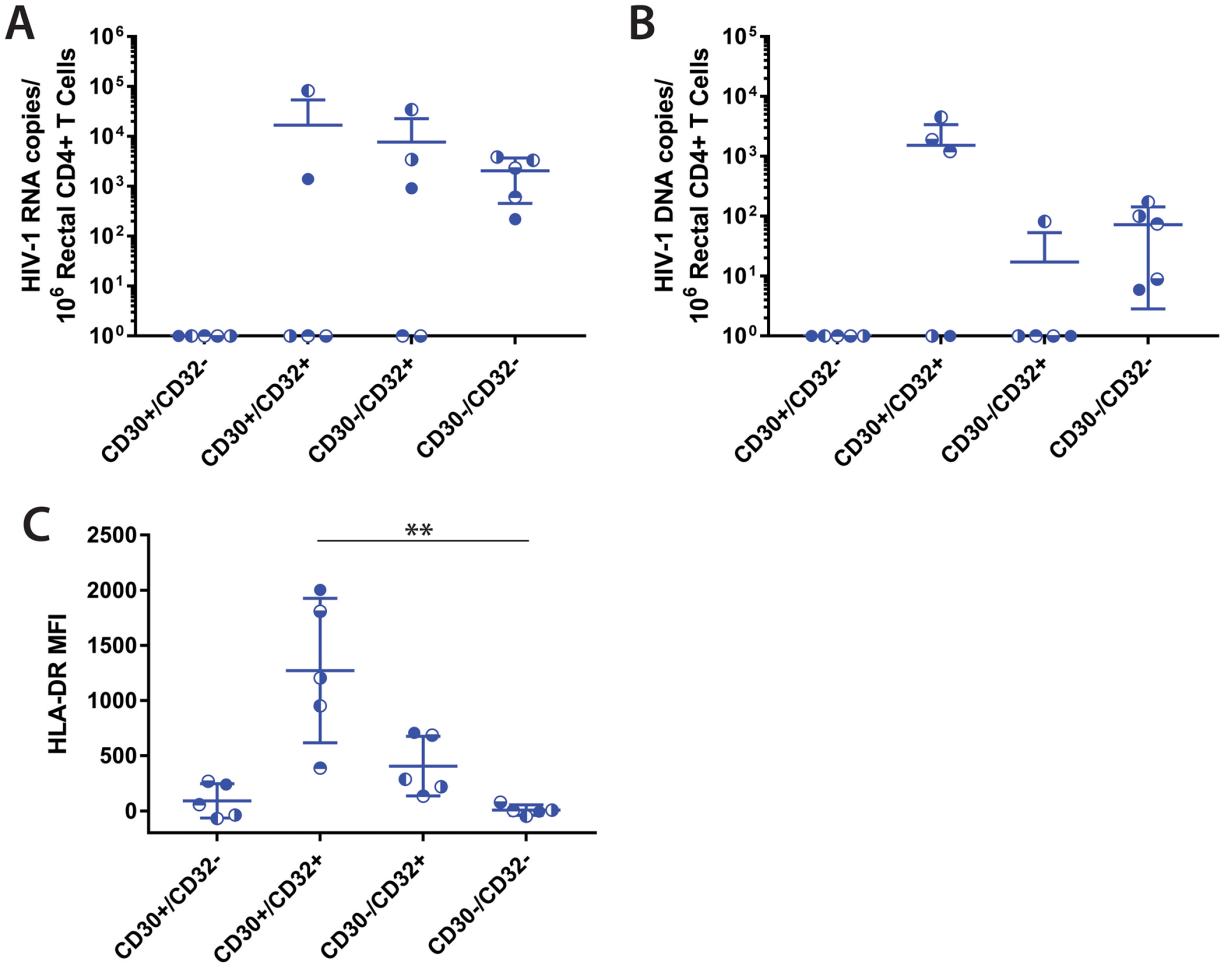
HIV-1 RNA and DNA quantification in rectal tissue-derived CD4^+^ T cell subsets. HIV-1 RNA and DNA levels for each CD4^+^ T cell subset (based on co-expression of CD30 and CD32) are shown in **A** and **B**, respectively. (**C**) A significantly higher mean HLA-DR MFI was observed in CD30^+^CD32^+^ cells compared with CD30^-^CD32^-^CD4^+^ T cells (P = 0.004 by Friedman test with Dunns correction for multiple comparisons). In contrast, no significant intergroup differences were observed in cell-associated HIV-1 RNA or DNA between cohorts. Bars represent mean ± standard deviation.

We obtained rectal tissue from five ART-suppressed individuals for phenotypic analysis and flow sorting of CD4^+^ T cells into CD30^+^, CD32^+^, CD30^+^CD32^+^, and remaining CD30^-^CD32^-^ subsets (S3 Fig & S3 Table) and incorporated CD13 into the staining panel of two donors to ascertain potential non-lymphocyte cell contamination (of note, CD14 is not expressed in gut-associated monocytes). Overall, we detected HIV-1 DNA and RNA in all of the CD30^-^CD32^-^CD4^+^ lymphocytes but none in the CD30^+^CD32^-^ population. HIV-1 RNA and DNA were intermittently detected in the CD30^+^/CD32^+^ and CD30^-^/CD32^+^ subsets, at times, 1–2 log_10_ higher than the CD30^-^CD32^-^ CD4^+^ T cells (Fig 3A and 3B). CD4^+^ T cells had significantly higher expression levels of HLA-DR within the CD32 expressing subpopulations compared to CD30^+^CD32^-^ and CD30^-^CD32^-^ cells (Fig 3C), and up to 42% of HLA-DR^+^CD30^+^CD32^+^ CD4^+^ T cells co-expressed CD13 compared to <11% of CD30^+^CD32^-^ cells. No significant differences in HIV-1 RNA or DNA were observed between cell subsets obtained from the gut samples. It is possible that the extensive processing and collagenase treatment used to isolate cells from GALT samples may have altered surface phenotypes. Therefore, we proceeded to examine tissue samples directly using direct mRNA staining.

### HIV-1 RNA and CD30 mRNA are co-localized in human gut tissues

**Fig 4:**
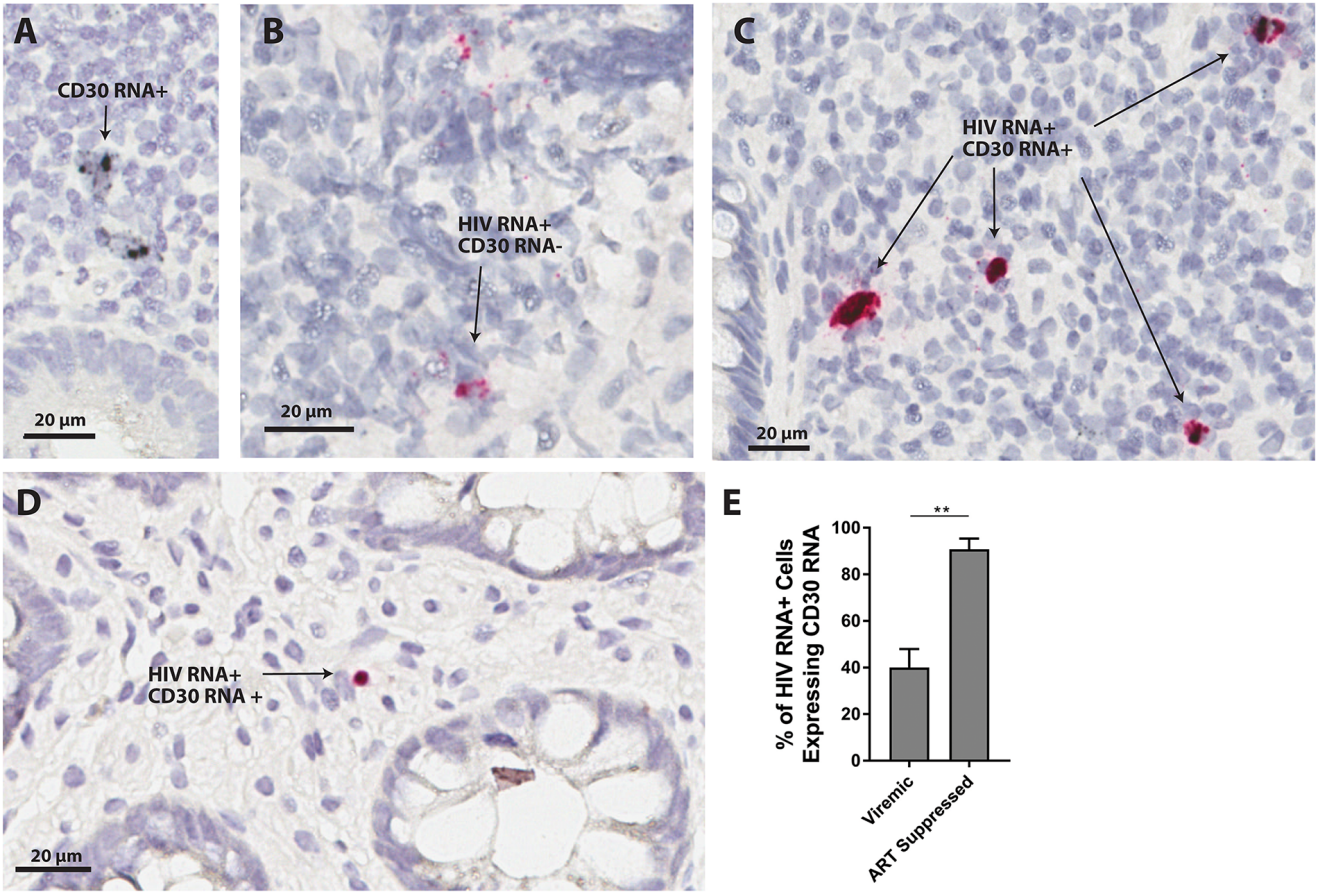
Cell-associated HIV-1 RNA and CD30 RNA co-localize in human rectal and ileal tissue from HIV-1-infected individuals. *In situ* chromogenic hybridization staining of HIV-1 RNA (pink) and CD30 RNA (black) was performed in ileum and rectum from HIV-1-infected individuals. Less than 0.1% of uninfected GALT cells expressed CD30 RNA (**A**). Although HIV-1 RNA^+^ staining alone was identified in sections from GALT from viremic HIV-1-infected individuals (n = 3) (**B**), 33% of HIV-1 RNA^+^ cells also expressed CD30 (**C**). In GALT from ART-suppressed individuals (n = 3), 88% of all HIV-1 RNA positive cells co-expressed CD30 (**D**). Percentages of HIV-1 RNA^+^ expressing CD30 RNA are shown in (**E**). 308 HIV-1 RNA^+^ cells were observed in sections from all viremic samples and 25 RNA^+^ cells were observed in tissues sections from suppressed individuals. Nearly all cells with high levels of CD30 RNA also expressed HIV-1 RNA, and minimal background CD30 RNA staining was observed in each field.

To determine the single-cell co-localization of HIV-1 and CD30 mRNA transcriptional activity in tissue, we performed *in situ* hybridization (ISH) on tissue samples obtained from ileum and rectum of HIV-1-infected individuals on (n = 6) and off (n = 3) ART, and one aviremic HIV-1 controller (S1 Table). In HIV-1-uninfected control tissue samples, <0.01% of all gut-associated lymphoid tissue (GALT) cells expressed CD30 RNA (Fig 4). However, the observed CD30 expression was almost exclusively co-localized with HIV-1 RNA in gut samples from both viremic and ART suppressed individuals (Fig 4). Interestingly, we found a significantly higher percentage of co-localization of CD30 and HIV-1 RNA in ART-suppressed individuals (88% of all HIV-1 RNA expressing cells in gut tissues expressed CD30) compared with co-localization in viremic participants (32.5%; P = 0.008).

### Quantitative viral outgrowth

In order to determine whether or not CD4^+^ T cells expressing CD30 harbour replication competent virus, we sorted PBMC from eight additional ART-suppressed individuals and performed traditional quantitative viral outgrowth assays (qVOA) incorporating serial dilutions of CD30^+^ T cell subsets. We were only able to detect positive p24 in CD30^-^CD32^-^CD4^+^ T cells in two of eight participant samples (total input cells ranged from 3 to 10 million) after 21 days of co-culture [infectious units per million cells (IUPM) of 0.11 and 0.23) We were not able to detect p24 in any wells containing CD30^+^ cells, but experiments were limited by the very small numbers of cells that could be recovered and incorporated in the qVOA experiments (< 1,000 input cells/well).

### Brentuximab vedotin reduces total cell-associated HIV-1 DNA in PBMC

**Fig 5:**
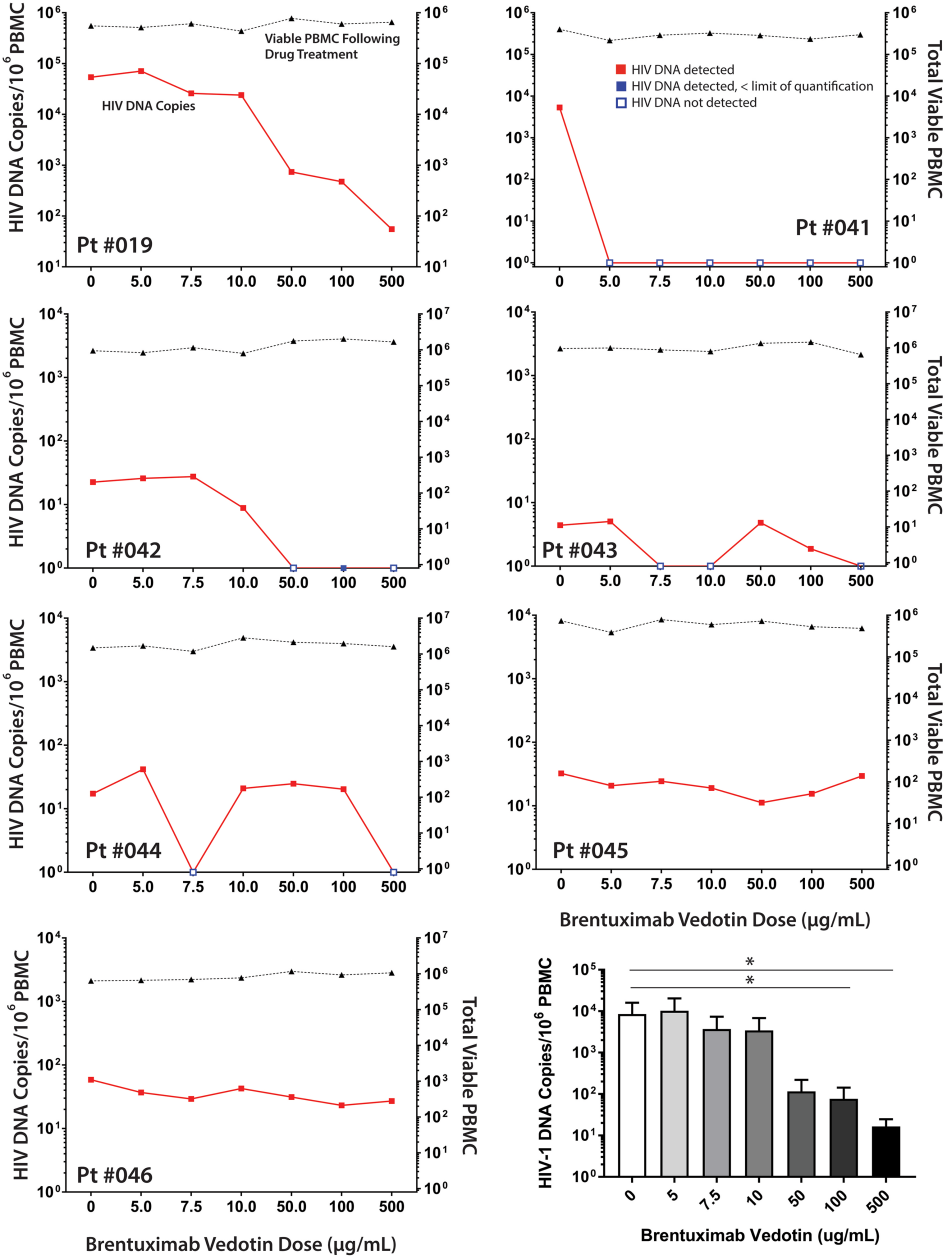
Anti-CD30 therapy reduces total HIV-1 DNA burden *ex vivo*. Following *ex vivo* culture of PBMC in the presence of antiretroviral drugs (raltegravir, 3TC) and brentuximab vedotin for 5 days, we observed a significant reduction in the mean total cell-associated HIV-1 DNA from seven ART-suppressed individuals, despite no change in viable cell numbers. Significant overall decreases were found within samples at the higher input concentrations (100μg/ml p = 0.0469, 500μg/ml p = 0.0390). Significant intergroup differences were determined using paired, nonparametric Friedman tests incorporating Dunn's tests for multiple comparisons. All experimental wells with and without brentuximab vedotin were subject to the same input cell number and culture conditions.

Brentuximab vedotin, an anti-CD30 antibody-drug conjugate approved for the treatment of various haematological malignancies in which CD30 is over expressed, has shown efficacy in reducing the burden of CD30^+^ malignant cells *in vivo* [10]. In preliminary experiments, we observed a 43% to 80% decrease in the percentages of CD4^+^ T cells expressing CD30 obtained from three ART-suppressed donors following 48 hours of *ex vivo* bretuximab vedotin exposure (10μg/ml) compared with parallel experimental wells with no antibody-drug conjugate (S4 Fig). Next, we exposed PBMC obtained from seven HIV-1-infected individuals on suppressive ART to various concentrations of brentuximab vedotin. Following culture of PBMC in the presence of antiretroviral drugs (raltegravir, 3TC) and brentuximab vedotin for 5 days, we observed a significant reduction in the mean total cell-associated HIV-1 DNA within samples at the higher input concentrations (100μg/ml p = 0.047, 500μg/ml p = 0.039; Fig 5). However, no reduction in the viability of total input PBMC was observed, suggesting that brentuximab vedotin may have selectively depleted CD30^+^ cells enriched in HIV-1 DNA. Overall, variations in DNA levels in no-ADC control wells were observed between participant samples. However, participants were not chosen based on baseline HIV reservoir size and all experimental wells, including those with and without brentuximab vedotin were exposed to the same input cell number and culture conditions.

### Undetectable cellular HIV-1 levels in an individual treated with brentuximab vedotin

Successful use of the brentuximab vedotin for refractory lymphomas has been described in two HIV-1-infected patients[26], but assessments of viral reservoir size after therapy have not been reported. We identified an individual who lacked detectable HIV-1 in cells after receiving six cycles of brentuximab vedotin for anaplastic and cutaneous T-cell lymphomas. The patient had low-level plasma viremia detectable after conventional chemotherapy (<400 RNA copies/ml), but no HIV-1 was detected in purified CD4^+^ T cells using sensitive real-time PCR assays for cell-associated RNA or DNA, and no plasma virus was detected by clinical viral load assays after six cycles of brentuximab vedotin. Unfortunately, the individual passed away from central nervous system tumor recurrence, and further longitudinal sampling or tissue collection was not possible. Furthermore, cells were not able to be collected prior to initiation of brentuximab vedotin. Nonetheless, this finding was unexpected as we have observed detectable HIV-1 DNA and RNA in PBMC from all other HIV-1-infected individuals in a cohort of 15 patients who completed multiple cycles of systemic chemotherapy (not including Brentuximab) for a variety of solid organ and hematological malignancies, as we have previously reported [27] (CD4^+^ T cell-associated HIV-1 DNA increased from a mean of 2,204 copies/10^6^ cells to 43,961 copies/10^6^ cells following completion of cancer chemotherapy in 10 individuals with pre- and post-chemotherapy time-points available). The HIV-1 DNA and RNA assays had a detection limit of <15 copies/10^6^ CD4^+^ T cells given the available number of input cells for this individual.

## Discussion

Previous studies have shown that higher levels of sCD30 are associated with HIV-1 disease progression [4,5,8,13,14,15,16,17,18,19,20]. However, the relationship between surface CD30 expression, sCD30 and HIV-1 burden in ART suppressed individuals and HIV controllers, is currently unknown. Here, we demonstrate that cell-associated HIV-1 RNA is significantly enriched in CD4^+^ T cells expressing CD30, a member of the tumor necrosis factor receptor superfamily, and this effect was observed in many, but not all, individuals. These findings were observed in several HIV-1 infected donor groups, regardless of whether or not the participants were receiving suppressive ART. HIV-1 DNA was also enriched in CD4^+^ lymphocytes expressing CD30 from some individuals, with HIV-1 DNA exclusively detected in CD30^+^ cells from one participant. When present, the magnitude of nucleic acid enrichment within CD30^+^CD4^+^ T cells was higher than previously reported for peripheral blood CD4^+^ T cells co-expressing PD-1 alone, or PD-1, TIGIT and LAG-3 (<1 log_10_ fold change) [28], and roughly similar to the frequency of lymph node-derived PD-1^+^CD4^+^ T cells that harbor inducible HIV-1 and total HIV-1 DNA burden in peripheral blood CD32^+^CD4^+^ T cells as previously described [29,30]. Overall, our results suggest that CD30 may be a marker of residual, transcriptionally active HIV-1 infected cells in setting of suppressive ART.

CD30 was found to be primarily expressed on peripheral blood CD4^+^ T_TM/EM_ subsets in HIV-1 infected donors. Although central memory T cells (T_CM_) have been shown to be a major peripheral blood reservoir of HIV [31], T_EM_ and T_TM_ subsets contribute significantly to the amount of cell-associated HIV-1 RNA and DNA in blood, and a majority of viral nucleic acid found in GALT, a major anatomic site of HIV persistence [32,33]. In addition, a recent study of six individuals on suppressive ART demonstrated that T_EM_ cells contained the largest proportion of intact HIV sequences [34]. Given the possibility that CD30 expression on CD4^+^ T_EM_ and T_TM_ subsets may be due to cellular activation, we sought to clarify if we were simply selecting a highly activated portion of CD4^+^ T cells, rather than a potential marker of viral infection. Encouragingly, the majority of activated T cells in all individual samples in our study did not co-express CD30. Furthermore, it is established that viremic individuals have higher levels of immune activation than individuals on ART [35]. In contrast, we observed that individuals on or off ART have similar frequencies of CD30^+^CD4^+^ T cells. This observation, in addition to our finding that <1% of CD4^+^ T cells, on average, were CD30 positive, suggest that CD30 is not a marker commonly expressed on activated T cells in the setting of HIV-1 infection, and may be due to more specific viral cellular stress responses.

Results from the gut *in-situ* hybridization studies also support the conclusion that HIV-1 RNA is enriched in CD30-expressing cells in individuals on suppressive ART. Although we were unable to differentiate between CD4^+^ T cells and tissue-resident macrophages or dendritic cells, nearly all CD30 mRNA^+^ cells co-expressed HIV-1 RNA, and a larger majority of HIV-1 RNA^+^ cells expressed high levels of CD30 mRNA. We did not find significant differences between HIV-1 RNA and DNA levels determined by qPCR in sorted CD30^-^ and CD30^+^ CD4^+^ T cell subsets isolated from fresh rectal tissue in ART-suppressed individuals, but the sample size was small and the number of CD32^+^ and/or CD30^+^ cells that could be recovered was often low. It is possible that gut tissue collagen digestion and processing influenced transcriptional regulation or surface presentation of CD30 and other surface markers, a phenomenon which has been previously described for other surface proteins [36]. The highest levels of HIV-1 RNA and DNA from rectal tissue-derived cells were obtained from cells co-expressing CD30 and CD32. Interestingly, this subset had the highest HLA-DR expression and greatest frequency of CD13 cells, suggesting that there may have been some degree of macrophage/myeloid carryover during tissue sorting. It is also possible that the HIV-1 RNA was enriched in activated gut-associated CD4^+^ T cells, as lymphocyte activation has been shown to be higher in GALT than peripheral blood [32]. As a result, further phenotypic characterization and study of HIV persistence in these tissue-derived cell subsets is needed.

Overall, our results suggest that CD30^+^ expression appears to be a marker of residual HIV-1 transcriptional activity in the setting of suppressive ART, at least in some individuals. However, several of our observations indicate that CD30 may also be a marker of more stable HIV-1 infection in the setting of ART or that CD30 expression waxes and wanes over time in infected cells. For example, sCD30 was only found to be higher in viremic individuals and sCD30 levels in ART-treated individuals were similar to those in uninfected controls. These data suggest that sCD30 may be increased as a result of persistent active viral production or excessive immune activation, whereas the surface expression of CD30 may represent a more stable, general HIV-related marker in both viremic and suppressed individuals. The mechanisms for the upregulation of CD30 in the setting of HIV-1 infection are unknown and warrant further investigation.

Finally, we demonstrated that *ex vivo* treatment with brentuximab vedotin, an antibody-drug conjugate that targets CD30, significantly reduced the total amount of HIV-1 DNA in PBMC obtained from seven HIV-infected individuals. These data suggest that CD30 may have a role as a potential HIV-1 therapeutic target, as it is highly correlated with residual HIV-1 transcriptional activity, even in individuals on suppressive ART, but is not expressed on a vast majority of otherwise healthy cells. Other cell surface markers that have previously been associated with HIV-1 enrichment (*e*.*g*. PD-1 or CD32) are expressed on a variety of non-T lymphocyte cell types which may make clearance of rare HIV-infected cells without significant off-target toxicities challenging. Furthermore, there are very few cytoreductive ADCs in clinical use, and these drugs are typically directed towards disease-specific targets [10].

We observed variation in responses to *ex vivo* brentuximab vedotin within this study, which is expected given the described variation within HIV-1 burden and CD30 expression between individuals in blood. It is possible that factors such as cellular HIV-1 DNA burden and the level of CD30 expression within individuals may influence the reservoir responses to brentuximab vedotin, and further studies involving concomitant latency reversal are warranted. While the observed loss of CD4^+^ T cell-associated HIV-1 DNA and RNA from the individual who received multiple cycles of brentuximab vedotin is only anecdotal, this case finding inspired the detailed *ex vivo* work of CD30 as a potential marker for HIV-1 infection as described above. Further identification of HIV-infected individuals receiving brentuximab vedotin for malignancy and detailed viral reservoir investigation are needed.

This study has several limitations. We observed that CD30 expression is significantly altered following freeze thaw procedures, and fresh blood draws were required. It is also highly possible that collagenase treatment of GALT samples altered surface phenotype, as previously described with other surface markers[36]. Additionally, the number of participants that could be recruited to the study was restricted and dependent on long cell-sorting time or the need for freshly reconstituted brentuximab vedotin. As a result, pre-selection of participants was limited and different individuals were included in each of the subsequent studies.

Overall, we noted high inter-participant variability in the HIV-1 reservoir measurements. However, variation between study participants is expected given the complex interplay between infection and expression of surface markers. At times, samples with low CD30 recovery from cell sorting resulted in the inability to detect HIV-1 RNA or DNA. Variability in the lower limits of detection has been previously documented using flow cytometric sorting of rare target cells [23]. Nevertheless, rather than exclude these samples, we included results from all participants to avoid biasing our results. In some instances, very high levels of HIV-1 RNA enrichment were observed in CD30^+^ T cells from a minority of participants. These high measurements may have partially been due to artefact from calculations involving very small denominators (*e*.*g*. small numbers of cells surveyed). However, we identified a very close relationship between HIV-1 RNA and CD30 mRNA expression in gut using *in situ* hybridization studies which provides additional support using an independent method that CD30 expression is directly related to cellular HIV-1 burden and transcriptional activity. We did not measure CD30 expression following ADC treatment in the brentuximab vedotin dose response experiments, but did observe large reductions in the percentage of CD4^+^ T cells expressing CD30 in the presence of ADC in preliminary experiments performed on samples from three ART-suppressed individuals. While this reduction may represent ADC-targeted cell killing, CD30 staining may also have been affected by steric interference with ADC-bound receptor or receptor downregulation.

We were unable to detect positive viral outgrowth in experiments including blood CD4^+^ T cells expressing CD30. This is likely due to limitations in the traditional qVOA when using very low numbers of input cells (in some cases, less than 100 cells per well for CD30^+^ subsets), and may or may not reflect the potential for CD30 expressing cells to harbor replication competent virus. Further studies involving methods, such as whole genome sequencing, may prove more useful in estimating intact proviral burden within CD30^+^ subsets.

In conclusion, we describe the enrichment of HIV-1 RNA in CD30^+^ CD4^+^ T cells from HIV-1 infected individuals on suppressive ART. Furthermore, a large proportion of total CD4^+^ T cell-associated HIV-1 RNA is found within CD30 expressing cells from suppressed and viremic individuals, and bretuximab vedotin is capable of reducing HIV-1 DNA burden. Further investigation is warranted to evaluate the stability of CD30 following HIV-1 infection and the development of latency, and to determine the efficacy of CD30 as a potential therapeutic target.

## Methods

### Study population and peripheral blood mononuclear cell collection

In total, 43 HIV-1-infected and ten HIV-uninfected participants ≥18 years of age were enrolled. Clinical data obtained included history of ART, viral load measurements, and CD4^+^ T cell counts, race/ethnicity, age, and detailed medical histories. Individuals on or off ART were included in the study. Leukapheresis was performed through the UCSF SCOPE cohort for the collection of >1 billion PBMC used in cell sorting and viral outgrowth assays (VOA).

### Ethics statement

The study was approved by the UCSF Committee for Human Research and the Dana-Farber/Harvard Cancer Centres Institutional Review board. All volunteers provided written informed consent.

### Peripheral blood cell isolation and rectal tissue processing

Peripheral blood mononuclear cells were separated from whole blood using density centrifugation over Histopaque (Sigma). Isolation of CD4^+^ T cells was then performed using EasySep Human CD4^+^ T Cell Enrichment Kits (Stem Cell Technologies), following the manufacturer’s protocol. For rectal tissue, to remove the epithelium and epithelial cells, 30 tissue biopsy tissue pieces were incubated in pre-warmed buffer containing 10mM DTT, 5mM EDTA, 10mM HEPES and 5% FBS for 20 minutes at 37°C under continuous rotation, vortexed and the supernatant aspirated. Tissue pieces were then incubated for a second time in the same pre-warmed buffer for a further 20 minutes at 37°C under continuous rotation. The sample was again vortexed, and then rinsed for 20 minutes at 37°C under continuous rotation with RMPI, 10mM HEPES and 5% FBS. To then isolate lymphocytes from the lamina propria, a second, pre-warmed buffer containing RPMI, 10mM HEPES, 7.5μg/ml DNAse and 5% FBS was applied to the tissue pieces, and these were again incubated for 20 minutes at 37°C under continuous rotation. The samples were then vortexed, and aspirated numerous times with a blunt 20G needle until tissue was viably broken down. The cells were then rinsed twice with RMPI, 10mM HEPES and 5% FBS and passed through a 70μm cell strainer, pelleted and stained as described below for fluorescent activated sorting.

### Antibody staining and fluoresce activated cell sorting

CD4^+^ T cells were stained in PBS with either Brilliant Violet 605-conjugated anti-CD4 (OKT4) (Biolegend), allophycocyanin (APC)-conjugated anti-CD30 (BY88) (Biolegend) and fluorescein isothiocyanate (FITC)-conjugated anti-CD3 (SK7) (BD Biosciences) for sorting only, or phycoerythrin (PE)-Cy7-conjugated anti-CCR7 (3D12) (BD Biosciences), Brilliant Violet 711-conjugated anti-CD3 (UCHT1) (BD Biosciences), Brilliant Violet 421-conjugated anti-PD-1 (EH12.1) (BD Biosciences), Brilliant Violet 650-conjugated anti-CD4 (SK3) (BD Biosciences), alexa Fluor 700-conjugated anti-HLA-DR (G46-6) (BD Biosciences), PE-conjugated anti-CD38 (HB7) (BD Biosciences), fluorescein isothiocyanate (FITC)-conjugated anti-CD69 (L78) (BD Biosciences), Qdot 605-conjugated anti-CD8 (3B5) (Life Technologies), PE-Texas Red-conjugated anti-CD45RA (MEM-56) (Life Technologies), allophycocyanin (APC)-conjugated anti-CD30 (BY88) (BioLegend) and LIVE/DEAD Fixable Aqua Dead Cell Stain Kit (ThermoFisher Scientific) for CD4^+^ CD30^+^ T cell phenotypical analysis. For the rectal tissue samples, cells were stained with LIVE/DEAD Fixable Aqua Dead Cell Stain Kit (ThermoFisher Scientific), PE-CF594-conjugated anti-CD45RA (HI100) (Fisher Scientific), Brilliant Violet 711-conjugated anti-CD4 (SK3) (BD Biosciences), Brilliant Violet 650-conjugated anti-CD3 (SP34-2) (BD Biosciences), allophycocyanin (APC)-conjugated anti-CD32 (FUN-2) (Biolegend), PE-conjugated anti-CD30 (BERH8) (BD Biosciences), APC-H7-conjugated anti-HLA-DR (L243) (BD Bioscience), Brilliant Violet 586-conjugated anti-CD13 (WM15) (BD Biosciences) and Brilliant Violet 421-conjugated anti-CD38 (HIT2) (Fisher Scientific).

Cells were then analyzed and sorted on a BD FACS ARIA II (BD Biosciences), or analyzed on a LSR-II (BD Bioscience). Single stained beads (Life Technologies) were used for software-based compensation. During some sorts, data for phenotyping was also acquired on all events and analyzed in FlowJo V10 (TreeStar). Examples of gating strategies are shown in S1 & S3 Figs.

### Quantification of cell-associated HIV-1 RNA and DNA

Purification of HIV-1 DNA and RNA from sorted cell populations was achieved using a Qiagen AllPrep DNA/RNA mini Kit, and following the manufacturer’s standard protocol, with an additional DNAse treatment (QIAgen). Given the small number of CD30^+^ cells that could be obtained from flow sorting, these cell fractions were spiked into uninfected carrier PBMC to maximize HIV-1 DNA and RNA recovery and normalize extraction efficiency between CD30^+^ and CD30^-^ CD4^+^ T cell populations. Spiking rare cells also allowed for the input of similar amounts of RNA into each PCR reaction. Quantitative PCR was performed to determine the levels of HIV-1 cell-associated RNA (caRNA), proviral DNA (pvDNA), and CCR5 in each subgroup. CCR5 was used to calculate assay cell concentration and extraction efficiency. Primer pairs and probe sequences were used as described in [24,37]. Briefly, the same primer and probe sequences were used for both total HIV-1 DNA and unspliced RNA and sit near the Gag-LTR junction, a highly conserved region among all group M HIV-1 sequences. PCR reactions were performed on an ABI OneStep qPCR machine (Applied Biosystems) using either the ABI TaqMan Universal PCR Master Mix for DNA or the ABI TaqMan Fast Virus 1-Step Master Mix for RNA for up to 45 cycles as we have previously described [24].

### Determination of soluble CD30

Plasma from EDTA anticoagulant blood was isolated by density centrifugation followed by an additional spin and frozen until all donors had been recruited. Plasma was then thawed, and analysed using Human CD30 (soluble) Elisa Kit (Life Technologies) following the manufacturer’s standard protocol.

### Cell culture and antibody-drug conjugate treatment

PBMC were cultured at 1 x 10^6^ cells/mL in RPMI medium (Life Technologies) supplemented with 10% heat-inactivated FBS (Gemini BioProducts), 100IU/ml penicillin and 100μg/ml streptomycin (Gemini BioProducts). Where required, Brentuximab Vedotin (Seattle Genetics) was added to cells at various concentrations. All cell cultures were performed in the presence of antiretroviral treatment [raltegravir (40nM), and efavirenz (8nM) or T20 (20ng/mL] (Selleck) and IL-2 (500ng/mL) (Peprotech). Cells were cultured for five days, after which analysis by flow cytometry, DNA extraction, and quantitative PCR were performed, as described above.

### *In situ* hybridization (ISH) of ileal and rectal tissue

Existing gut tissue was obtained through the UCSF SCOPE cohort by colonoscopy. Three millimeter sections were obtained from the gut and/or ileum (by jumbo forceps) of HIV-1-infected individuals on or off ART. Informed consent was obtained from all participants under the approval of the UCSF Committee on Human Research. Tissue was promptly preserved in 4% paraformaldehyde before paraffin embedding and sectioning for ISH. RNA-ISH was performed using RNAscope branched-DNA technology distributed by Advanced Cell Diagnostics (ACD), Newark, CA. The RNAscope assay was performed on 4-micron thick sections of FFPE human gut tissue (ileal and rectal) using the RNAscope 2.5 HD duplex kit. RNAscope probes targeting HIV-1 RNA, and the anti-sense sequence of the Hs-TNFRSF8 gene (CD30) were developed by ACD and used according to the manufacturer’s recommendations. The HIV-1 infected ACH2 T cell line and HIV-negative rectal tissues were used as positive and negative controls, respectively. Sections were stained in multiplex for CD30 and HIV-1 RNA species and analyzed by bright-field microscopy. Whole slide digital images were obtained using the Leica Aperio slide scanner and total HIV-1-RNA^+^ or HIV-1-RNA^+^/CD30-RNA^+^ cells were manually counted in digital images of each section of tissues. Signal intensity was used as a qualitative measure of the level of transcription where small-punctate signals are thought to represent a single transcript and larger-darker clusters are thought to represent multiple transcripts in close proximity [38].

### PCR T cell array

PBMC from an additional five individuals on suppressive ART were sorted into CD30^+^ and CD30^-^ subsets followed by direct cell lysis and reverse transcription of mRNA to cDNA using TaqMan Gene Expression Cells-to-CT Kit (Life Technologies/Ambion) following manufacturer protocols, with the exception of the final preamplification step. cDNA of the CD3 receptor complex, CD4, CD80, CD8α and reference gene (18S and GUSB) mRNA were then quantified using the ABI OnseStep-compatible Human T-Cell Receptor and CD3 Complex TaqMan Array (ThermoFisher) following manufacturer protocols. 2^-ΔΔCt^ values were calculate as described [39] comparing CD30^+^ and CD30^-^ CD4^+^ T cell subsets. B cells purified by negative magnetic bead selection (StemCell Technologies) were used as a control.

### Viral outgrowth assays

Following fluorescent cell sorting of CD4^+^ T cell subsets based on surface marker expression as described above, cells were incorporated into a standard quantitative viral outgrowth assay exactly as previously describe [40]. In brief, this assay uses irradiated PMBC blasts, PHA and IL2 to maximally stimulate input cells, and is considered a gold-standard for quantitative co-culture. If sufficient cells were recovered, 5-fold serial dilutions starting with one to two million input cells per well were performed in duplicate. HIV-1 p24 levels were then determined by ELISA (PerkinElmer, San Jose, CA) following 21 days of co-culture. A well was considered positive if p24 levels were significantly higher than background levels from control wells containing uninfected cells. IUPMs and confidence intervals were determined using an online calculator incorporating maximum likelihood statistics as described [41].

### Statistical methods

Significant intergroup differences were determined using rank Kurskal-Wallis of Friedman tests (depending on paired or unpaired data) incorporating Dunn's tests for multiple comparisons. Wilcoxon matched-pairs signed rank tests were used to determine statistical significant between CD30 intergroup, paired samples. (Graphpad Software, vs. 7).

## Supporting information

S1 Table Patient Demographics, HIV-1 disease status and antiretroviral therapy for HIV-1-infected participants. (DOCX)

S2 Table Experimental data for HIV-1 DNA and RNA PCR calculations. (DOCX)

S3 Table Absolute cell numbers obtained following fluorescence-activated cell sorting of rectal tissue samples. (DOCX)

S1 Fig Example of gating strategy used in flow cytometric and fluorescent activated cell sorting experiments. **(A)** Lymphocytes populations were first selected using forward and side scatter characteristics. Following this, the exclusion of doublets was performed by plotting cells on SSC-W versus SSC-H and similarly for FSC-W and FSC-H. Live and then CD3^+^ lymphocytes were selected, and further gated for CD4 expression. Following this CD4^+^ CD30^+^ T cells and CD30^-^CD4^+^ T cells were selected in individual gates and sorted (for sorting experiments). **(B)** For further phenotypical analysis, the same gating was applied as described in (A) and then extended, allowing the identification of CD30 expressing cells with CD45RA and CCR7 populations (T cell subsets), CD69 (early activation), CD38 and HLA-DR (Late activation) and PD-1 expression. **(C)** CD30 expressing cells (red) are then compared and contrasted to CD30 negative CD4^+^ T cell populations (Blue), shown together on the same plots. A fluorescence minus one for APC-conjugated anti-CD30 was included to establish CD30 gating.
(DOCX)

S2 Fig 2^-ΔΔCt^ values comparing mRNA from CD30^+^ versus CD30^-^ CD4^+^ T cell subsets obtained from five individuals on suppressive ART were determined by Taqman PCR gene array for CD3 complex, CD4, CD8 and CD4 genes. Of note, no CD80 mRNA could be detected in four samples (*) and no CD8α mRNA could be detected in three samples (†). Overall, 2^-ΔΔCt^ values of CD3 complex and CD4 mRNA were similar or higher comparing CD30^+^ and CD30^-^ CD4^+^ T cells. No CD3 complex, CD4 or CD8 mRNA could be detected from purified B cells obtained from an uninfected donor which served as a control. 2^-ΔΔCt^ values represent a function comparing CD30^+^ to CD30^-^ CD4^+^ T cell mRNA levels. A value of 1 represents no difference between populations within an individual sample and values greater than 1 indicate a greater number of mRNA transcripts.
(DOCX)

S3 Fig Example of gating strategy used in GALT sorting of CD4^+^ T cells to determine CD30 and CD32 expression. Lymphocytes populations were first selected using forward and side scatter characteristics. Following this, the exclusion of doublets was performed by plotting cells on FSC-W and FSC-A and similarly for SSC-W versus SSC-A. Live and then CD45^+^ lymphocytes were selected, and further gated for CD3^+^CD4^+^ T cells. Following this CD4^+^ CD30^+^, CD4^+^ CD32^+^, and CD4^+^ CD30^+^ CD32^+^ T cells were selected in individual gates and sorted. Further phenotypical information was collected on each T cell subset, including CD38, HLA-DR and CD13 expression.
(DOCX)

S4 Fig The use of the anti-CD30 cytotoxic antibody-drug conjugate, brentuximab vedotin (10 μg/mL), reduced the percentage of CD4^+^ T cells expressing CD30 after 48 hours of culture in the presence of ART (n = 3). While this reduction may represent ADC-targeted cell killing, CD30 staining may also have been affected by steric interactions with ADC-bound receptor or receptor downregulation.
(DOCX)
