# Supplementary material for: Increased HIV-1 transcriptional activity and infectious burden in peripheral blood and gut-associated CD4+ T cells expressing CD30
Source: PLoS Pathog. 2018 Feb 22;14(2):e1006856. doi: 10.1371/journal.ppat.1006856 (PMC5823470; doi:10.1371/journal.ppat.1006856)
Supplement: S1 Table — (DOCX) [file ppat.1006856.s001.docx]

| **S1 Table:** Patient Demographics, HIV-1 Disease Status and Antiretroviral Therapy for HIV-1-Infected Participants. | | | | | | | |  |  |
| --- | --- | --- | --- | --- | --- | --- | --- | --- | --- |
| **PID #** | **Donor**  **Group** | **Last HIV Viral Load (RNA copies/ml)** | **CD4** | **Gender** | **Ethnicity** | **Age** | **ART** | | **Source Tissue or Whole Blood (WB)** |

| 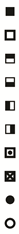 | 001 | Negative | - | - | Female | White | 35 | - | WB |
| --- | --- | --- | --- | --- | --- | --- | --- | --- | --- |
|  | 002 | Negative | - | - | Female | Asian | 42 | - | WB |
|  | 003 | Negative | - | - | Female | White | 32 | - | WB |
|  | 004 | Negative | - | - | Female | White | 24 | - | WB |
|  | 005 | Negative | - | - | Female | African American | 60 | - | WB |
|  | 006 | Negative | - | - | Male | White | 42 | - | WB |
|  | 007 | Negative | - | - | Female | White | 34 | - | WB |
|  | 008 | Negative | - | - | Male | Hispanic | 36 | - | WB |
|  | 009 | Negative | - | - | Male | White | 41 | - | WB |
|  | 010 | Negative | - | - | Male | White | 36 | - | WB |
| 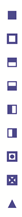 | 011 | Suppressed | <40^a^ | 733 | Male | African American | 65 | 3TC, EFV, RAL | WB |
|  | 012 | Suppressed | <40^a^ | 879 | Male to Female Transgender | African American | 55 | ABC/DTG/3TC | WB |
|  | 013 | Suppressed | <40^a^ | 348 | Male | White | 52 | FTC/TDF, DTG | WB |
|  | 014 | Suppressed | <40^a^ | 478 | Male | African American | 55 | FTC/TDF, DTG | WB |
|  | 015 | Suppressed | <40^a^ | 578 | Male | Native American | 38 | RPV/TDF/FTC | WB |
|  | 016 | Suppressed | <40^a^ | 858 | Male | Hispanic/Latino | 55 | FTC/TDF, DTG | WB |
|  | 017 | Suppressed | <40^a^ | 631 | Male | White | 61 | ABC/DTG/3TC | WB |
|  | 018 | Suppressed | <40^a^ | 1056 | Male | White | 50 | TDF/FTC, DRV/r | WB |
|  | 019 | Suppressed | <40^a^ | 524 | Male | African American | 59 | EFV/TDF/FTC | WB |
| 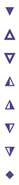 | 020 | Suppressed | <40^a^ | 568 | Male | White | 64 | FTC/TDF, ATV/r | WB |
|  | 021 | Suppressed | <40^a^ | 603 | Male | White | 60 | EFV/TDF/FTC, RAL | WB |
|  | 022 | Suppressed | <40^a^ | 798 | Male | White | 53 | TAF/FTC/EGV/c | WB |
|  | 023 | Suppressed | <40^a^ | 393 | Male | African American | 56 | FTC/TDF, ATV/r, RAL | WB |
|  | 024 | Suppressed | <40^a^ | 751 | Female | African American | 49 | ABC/3TC, ATV/r | WB |
|  | 025 | Suppressed | <40^a^ | 1073 | Female | African American | 61 | RPV/TDF/FTC | WB |
|  | 026 | Suppressed | ^b^ | 824 | Male | White | 78 | FTC/TDF, ETV | WB |
|  | 027 | Suppressed | <40^a^ | 518 | Male | White | 62 | ABC/3TC, ETV, RAL | WB |
| 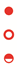 | 028 | Viremic | 9436 | 308 | Male | African American | 52 | None | WB/Rectum |
|  | 029 | Viremic | 348798 | 332 | Male to Female Transgender | African American | 41 | None | WB |
|  | 030 | Viremic | 5418 | 784 | Male | White | 44 | None | WB |
| 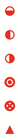 | 031 | Viremic | 7681 | 472 | Male | White | 48 | None | WB |
|  | 032 | Viremic | 46986 | 423 | Male | African American | 48 | None | WB |
|  | 033 | Viremic | 308383 | 308 | Male | White | 36 | None | WB |
|  | 034 | Viremic | 9638 | 406 | Male | Mixed Race/Multiracial | 33 | None | WB |
|  | 035 | Viremic | ^c^ | 194 | Male | White | 62 | None | WB |
|  | 036 | Viremic | ^c^ | 402 | Male | Asian | 30 | None | WB |
| 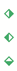 | 037 | HIV Controller | 461 | 1007 | Male | African American | 40 | None | WB |
|  | 038 | HIV Controller | <40^a^ | 1126 | Male | African American | 62 | None | WB |
|  | 039 | HIV Controller | <40^a^ | 845 | Male | African American | 80 | None | WB |
|  | 040 | Suppressed | <40^a^ | 743 | Female | White | 33 | TAF/FTC/EGV/c | WB |
|  | 041 | Suppressed | <40^a^ | 898 | Male | White | 68 | 3TC, DRV/r, DTG | WB |
|  | 042 | Suppressed | <40^a^ | 949 | Male | White | 42 | ABC/DTG/3TC | WB |
|  | 043 | Suppressed | <40^a^ | 1257 | Male | Hispanic/Latino | 42 | ABC/DTG/3TC | WB |
|  | 044 | Suppressed | <40^a^ | 615 | Male | White | 66 | ABC/DTG/3TC | WB |
|  | 045 | Suppressed | <40^a^ | 824 | Male | White | 61 | ABC/DTG/3TC | WB |
| 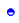 | 046 | Suppressed | <40^a^ | 299 | Male | Asian | 60 | FTC/RPV/TAF | WB/Rectum |
|  | 047 | HIV negative | - | 301 | Male | Hispanic/Latino | 46 | - | Rectum |
|  | 048 | HIV Controller | <40^a^ | 411 | Male | Mixed Race/Multiracial | 59 | None | Rectum |
|  | 049 | Viremic | 69004 | 198 | Male | African American | 54 | None | Rectum |
|  | 050 | Viremic | 91199 | 208 | Male | White | 50 | None | Rectum |
|  | 051 | Suppressed | <40^a^ | 247 | Male | White | 54 | TDF/FTC, DTG | Rectum / Ileum |
|  | 052 | Suppressed | <40^a^ | 478 | Male | White | 59 | FTC/TDF, DRV/r, DTG | Rectum / Ileum |
|  | 053 | Suppressed | <40^a^ | 226 | Male | Mixed Race/Multiracial | 67 | DTG/ABC/3TC | Rectum / Ileum |
| 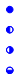 | 054 | Suppressed | <40^a^ | 589 | Male | White | 57 | DTG/ABC/3TC | Rectum |
|  | 055 | Viremic | 5969 | 497 | Male | Mixed Race/Multiracial | 49 | None | Rectum |
|  | 056 | Suppressed | <40^a^ | 402 | Male | African American | 61 | EFV/TDF/FTC | Rectum |
|  | 057 | Suppressed | <40^a^ | 556 | Male | White | 63 | DTG, RPV | Rectum |

| ABC = abacavir; ATZ/r = ritonivari boosted atazanvir; DTG = dolutegravir; 3TC = lamivudine; TDF = tenofovir; FTC = emtricitabine; EFV = efavirenz; RAL = raltegravir; RPV = rilpivarine; ETV = etravirene; EVG/c = cobicistat boosted elvitegravir; TAF = tenofovir alafenamide, DRV/r = ritonovir boosted darunavir  ^a^ Plasma HIV RNA copies either not detected or <40 copies/ml  ^b^ Viral load < assay detection as per outside provider  ^c^ Viral load not available |
| --- |
