# Supplementary material for: Increased HIV-1 transcriptional activity and infectious burden in peripheral blood and gut-associated CD4+ T cells expressing CD30
Source: PLoS Pathog. 2018 Feb 22;14(2):e1006856. doi: 10.1371/journal.ppat.1006856 (PMC5823470; doi:10.1371/journal.ppat.1006856)
Supplement: S2 Table — (DOCX) [file ppat.1006856.s002.docx]

| S2 Table: Experimental Data for HIV-1 DNA and RNA PCR Calculations | | | | | | | | | | | | | | |
| --- | --- | --- | --- | --- | --- | --- | --- | --- | --- | --- | --- | --- | --- | --- |
| PID # | **Subset** | **Input Cell**  **No.** | **Cell No. +**  **HIV neg spike cells** | **Cell No.**  **Ratio** | **CCR5 (1/10 dilution)** | **Output**  **Cell No.** | **HIV DNA per 10 µL** | **Total DNA**  **Extraction  Volume** | **Total**  **HIV DNA in  Extraction** | **HIV**  **DNA/10^6^ Cells** | **HIV RNA per 10 µL** | **Total RNA**  **Extraction Volume** | **Total**  **HIV RNA in Extraction** | **HIV**  **RNA/10^6^ Cells** |
| 011 | CD30- | 9000000 | 9000000 | 1 | 185400.5 | 927002.38 | 17.46 | 100 | 174.61 | 188.36 | 6.28 | 60 | 37.69 | 40.65 |
|  | CD30+ | 2806 | 4002806 | 0.000701 | 125400.1 | 439.53 | 26.01 | 100 | 260.07 | 591701.1 | 56.79 | 60 | 340.76 | 775273.27 |
| 012 | CD30- | 1096047 | 1096047 | 1 | 33965.7 | 127371.37 | 9.01 | 75 | 67.54 | 530.29 | 664.55 | 60 | 3987.31 | 31304.6 |
|  | CD30+ | 69727 | 1069727 | 0.065182 | 66356.5 | 16219.7 | 0 | 75 | 0 | 0 | 102.03 | 60 | 612.21 | 37744.82 |
| 013 | CD30- | 3503086 | 3503086 | 1 | 39260.98 | 147228.66 | 11.07 | 75 | 83.06 | 564.16 | 7.38 | 60 | 44.3 | 300.86 |
|  | CD30+ | 801 | 1000801 | 0.0008 | 28197.45 | 84.63 | 0 | 75 | 0 | 0 | 0 | 60 | 0 | 0 |
| 014 | CD30- | 1182059 | 1182059 | 1 | 259918.4 | 974694.14 | 8.21 | 75 | 61.58 | 63.18 | 494.34 | 60 | 2966.03 | 3043.04 |
|  | CD30+ | 6586 | 6586 | 1 | 31947.38 | 119802.69 | 0 | 75 | 0 | 0 | 85.25 | 60 | 511.47 | 4269.28 |
| 015 | CD30- | 2200000 | 2200000 | 1 | 33353.03 | 125073.86 | 0 | 75 | 0 | 0 | 0.37 | 60 | 2.23 | 17.86 |
|  | CD30+ | 2680 | 1002680 | 0.002673 | 258520.5 | 2591.19 | 0 | 75 | 0 | 0 | 0.47 | 60 | 2.84 | 1097.54 |
| 016 | CD30- | 911986 | 911986 | 1 | 34019.71 | 127573.92 | 12 | 75 | 90.03 | 705.7 | 493.97 | 60 | 2963.83 | 23232.27 |
|  | CD30+ | 27 | 1000027 | 2.70E-05 | 99505.42 | 10.07 | 0 | 75 | 0 | 0 | 56.49 | 60 | 338.93 | 33641936 |
| 017 | CD30- | 4700000 | 4700000 | 1 | 165078.6 | 825393.05 | 14.99 | 100 | 149.88 | 181.58 | 129.16 | 100 | 1291.59 | 1564.82 |
|  | CD30+ | 1540 | 3001540 | 0.000513 | 147794.9 | 379.15 | 0 | 100 | 0 | 0 | 0 | 100 | 0 | 0 |
| 018 | CD30- | 7000000 | 7000000 | 1 | 136518.2 | 511943.37 | 46.08 | 75 | 345.58 | 675.04 | 1037.39 | 75 | 7780.43 | 15197.84 |
|  | CD30+ | 4880 | 1004880 | 0.004856 | 839477.3 | 15287.83 | 0.75 | 75 | 5.62 | 367.32 | 58.9 | 75 | 441.73 | 28893.94 |
| 019 | CD30- | 4000000 | 4000000 | 1 | 53510.81 | 160532.43 | 0 | 60 | 0 | 0 | 618.44 | 60 | 3710.67 | 23114.76 |
|  | CD30+ | 668 | 1000668 | 0.000668 | 24440.82 | 48.95 | 0 | 60 | 0 | 0 | 317.45 | 60 | 1904.68 | 38913420 |
| 020 | CD30- | 5000000 | 5000000 | 1 | 536122.6 | 2010459.7 | 10.3 | 75 | 77.24 | 38.42 | 290.29 | 60 | 1741.76 | 866.35 |
|  | CD30+ | 8 | 1000008 | 8.00E-06 | 54338.37 | 1.63 | 0 | 75 | 0 | 0 | 0 | 60 | 0 | 0 |
| 021 | CD30- | 5600000 | 5600000 | 1 | 106229.9 | 398362.24 | 86.29 | 75 | 647.17 | 1624.58 | 345.76 | 75 | 2593.17 | 6509.57 |
|  | CD30+ | 7304 | 1007304 | 0.007251 | 388432.3 | 10562.02 | 0 | 75 | 0 | 0 | 0 | 75 | 0 | 0 |
| 022 | CD30- | 10000000 | 10000000 | 1 | 149860.7 | 749303.63 | 0 | 100 | 0 | 0 | 1165.1 | 60 | 6990.62 | 9329.48 |
|  | CD30+ | 5400 | 10005400 | 0.00054 | 72970.08 | 196.91 | 0.51 | 100 | 5.11 | 25929.28 | 10.2 | 60 | 61.21 | 310866.62 |
| 023 | CD30- | 139223 | 139223 | 1 | 72441.16 | 271654.36 | 22.39 | 75 | 167.95 | 618.25 | 364.93 | 60 | 2189.59 | 8060.19 |
|  | CD30+ | 205383 | 1205383 | 0.170388 | 66067.97 | 42214.5 | 5.27 | 75 | 39.51 | 935.84 | 317.08 | 60 | 1902.49 | 45067.15 |
| 024 | CD30- | 10041281 | 10041281 | 1 | 39939.04 | 149771.41 | 9.92 | 75 | 74.41 | 496.8 | 30.36 | 60 | 182.16 | 1216.24 |
|  | CD30+ | 3535 | 1003535 | 0.003523 | 27047.65 | 357.29 | 3.16 | 75 | 23.69 | 66313.74 | 0 | 60 | 0 | 0 |
| 025 | CD30- | 4200000 | 4200000 | 1 | 132690.8 | 663453.82 | 0 | 100 | 0 | 0 | 0 | 60 | 0 | 0 |
|  | CD30+ | 995 | 4000995 | 0.000249 | 109890.7 | 136.64 | 0 | 100 | 0 | 0 | 98.55 | 60 | 591.28 | 4327170.2 |
| 026 | CD30- | 1978098 | 1978098 | 1 | 53705.46 | 201395.49 | 13.34 | 75 | 100.05 | 496.8 | 542.22 | 60 | 3253.32 | 16153.88 |
|  | CD30+ | 7511 | 1007511 | 0.007455 | 44310.06 | 1238.74 | 14.35 | 75 | 107.61 | 86866.99 | 8.95 | 60 | 53.69 | 43339.82 |
| 027 | CD30- | 860000 | 860000 | 1 | 33139.8 | 124274.25 | 18.05 | 75 | 135.37 | 1089.25 | 279.85 | 75 | 2098.89 | 16889.22 |
|  | CD30+ | 3455 | 1003455 | 0.003443 | 394526.3 | 5093.98 | 0.04 | 75 | 0.31 | 60.73 | 18.16 | 75 | 136.17 | 26732.51 |
| 028 | CD30- | 3000000 | 3000000 | 1 | 121718.3 | 365154.94 | 328.99 | 60 | 1973.91 | 5405.68 | 0 | 60 | 0 | 0 |
|  | CD30+ | 280000 | 1280000 | 0.21875 | 111332.7 | 73062.08 | 51.15 | 60 | 306.88 | 4200.23 | 192.12 | 60 | 1152.73 | 15777.42 |
| 029 | CD30- | 3000000 | 3000000 | 1 | 173400.9 | 867004.4 | 2165.2 | 100 | 21651.95 | 24973.29 | 10346.21 | 60 | 62077.24 | 71599.68 |
|  | CD30+ | 1496 | 3001496 | 0.000498 | 113710.4 | 283.38 | 6.05 | 100 | 60.55 | 213667.31 | 97.36 | 60 | 584.16 | 2061433.9 |
| 030 | CD30- | 1300000 | 1300000 | 1 | 45203.02 | 169511.32 | 18.44 | 75 | 138.32 | 816.02 | 20.91 | 60 | 125.47 | 740.19 |
|  | CD30+ | 217 | 1000217 | 0.000217 | 414651.2 | 337.35 | 0 | 75 | 0 | 0 | 8.37 | 60 | 50.21 | 148837.81 |
| 031 | CD30- | 8000000 | 8000000 | 1 | 277580.2 | 1387900.8 | 130.76 | 100 | 1307.64 | 942.17 | 1796.99 | 60 | 10781.92 | 7768.51 |
|  | CD30+ | 2000 | 1002000 | 0.001996 | 12090.64 | 120.67 | 0.62 | 100 | 6.18 | 51212.02 | 210.43 | 60 | 1262.61 | 10463738 |
| 032 | CD30- | 4200000 | 4200000 | 1 | 307730.1 | 1538650.3 | 710.49 | 100 | 7104.9 | 4617.62 | 5734.73 | 60 | 34408.35 | 22362.69 |
|  | CD30+ | 1211 | 4001211 | 0.000303 | 228850.4 | 346.32 | 0 | 100 | 0 | 0 | 160.96 | 60 | 965.77 | 2788681.4 |
| 033 | CD30- | 2300000 | 2300000 | 1 | 111417.5 | 417815.77 | 1875.74 | 75 | 14068.05 | 33670.47 | 3.91 | 60 | 23.48 | 56.2 |
|  | CD30+ | 12351 | 1012351 | 0.0122 | 183221.2 | 8382.59 | 67.76 | 75 | 508.17 | 60621.76 | 7714.6 | 60 | 46287.63 | 5521879.1 |
| 034 | CD30- | 6000000 | 6000000 | 1 | 480988 | 1803705.2 | 9.98 | 75 | 74.82 | 41.48 | 241.81 | 60 | 1450.84 | 804.37 |
|  | CD30+ | 9 | 1000009 | 9.00E-06 | 98264.34 | 3.32 | 0 | 75 | 0 | 0 | 37.98 | 60 | 227.88 | 68713031 |
| 035 | CD30- | 2260000 | 2260000 | 1 | 95210.01 | 476050.06 | 0 | 100 | 0 | 0 | 2949.11 | 60 | 17694.68 | 37169.78 |
|  | CD30+ | 5902 | 2005902 | 0.002942 | 64970.95 | 955.83 | 0 | 100 | 0 | 0 | 230.07 | 60 | 1380.45 | 1444245.1 |
| 036 | CD30- | 188393 | 188393 | 1 | 8974.73 | 33655.24 | 9.16 | 75 | 68.7 | 2041.29 | 73.94 | 60 | 443.62 | 13181.45 |
|  | CD30+ | 35 | 1000035 | 3.50E-05 | 169000.9 | 22.18 | 0 | 75 | 0 | 0 | 0 | 60 | 0 | 0 |
| 037 | CD30- | 3700000 | 3700000 | 1 | 182793.1 | 685474.29 | 0 | 75 | 0 | 0 | 7.6 | 65 | 49.4 | 72.06 |
|  | CD30+ | 2036 | 1002036 | 0.002032 | 31270.58 | 238.27 | 0 | 75 | 0 | 0 | 2.59 | 65 | 16.84 | 70668.15 |
| 038 | CD30- | 11000000 | 11000000 | 1 | 120255.2 | 450956.84 | 4.09 | 75 | 30.7 | 68.08 | 0 | 75 | 0 | 0 |
|  | CD30+ | 3089 | 1003089 | 0.003079 | 335543.4 | 3874.88 | 0 | 75 | 0 | 0 | 67.59 | 75 | 506.91 | 130818.55 |
| 039 | CD30- | 3000000 | 3000000 | 1 | 32877.02 | 98631.06 | 0 | 60 | 0 | 0 | 118.09 | 60 | 708.51 | 7183.47 |
|  | CD30+ | 274 | 1900274 | 0.000144 | 409913.2 | 177.32 | 0 | 60 | 0 | 0 | 45.71 | 60 | 274.24 | 1546637.7 |

| PID # | Subset | Input Cell  No. | Cell No. +  HIV neg spike cells | Cell No.  Ratio | CCR5  (1/10 dilution) | Output  Cell No. | HIV DNA  per 10 µL | Total DNA  Extraction  Volume | | Total  HIV DNA in Extraction | HIV  DNA/10^6^ Cells | HIV RNA  per 10 µL | Total RNA  Extraction Volume | Total  HIV RNA  in Extraction | HIV  RNA/10^6^ Cells |
| --- | --- | --- | --- | --- | --- | --- | --- | --- | --- | --- | --- | --- | --- | --- | --- |
| 063 | **CD30** | 4090 | 1004090 | 0.004073 | 604612.5 | 9235.4711 | 0 | | 75 | 0 | 0 | 0 | 60 | 0.00 | 0.00 |
|  | **CD32** | 12954 | 1012954 | 0.012788 | 687776.4 | 32983.194 | 0 | | 75 | 0 | 0 | 5.05 | 60 | 30.31 | 918.83 |
|  | **CD30&CD32** | 14179 | 1014179 | 0.013981 | 1016488 | 53292.28 | 0 | | 75 | 0 | 0 | 12.45 | 60 | 74.70 | 1401.77 |
|  | **Neg** | 1229500 | 2229500 | 0.551469 | 1028234 | 2126397.1 | 1.674262 | | 75 | 12.56 | 5.91 | 77.91 | 60 | 467.44 | 219.83 |
|  |  |  |  |  |  |  |  | |  |  |  |  |  |  |  |
| 047 | **CD30** | 899 | 1000899 | 0.000898 | 106303.9 | 358.05515 | 0 | | 75 | 0 | 0 | 0 | 60 | 0 | 0 |
|  | **CD32** | 8510 | 1008510 | 0.008438 | 84155.45 | 2662.9491 | 0 | | 75 | 0 | 0 | 0 | 60 | 0 | 0 |
|  | **CD30&CD32** | 717 | 1000717 | 0.000716 | 125989.4 | 338.51127 | 0.054393 | | 75 | 0.41 | 1205.12 | 0 | 60 | 0 | 0 |
|  | **Neg** | 558121 | 1558121 | 0.358201 | 180281.7 | 242164.31 | 2.408821 | | 75 | 18.07 | 74.60 | 24.51 | 60 | 147.09 | 607.39 |
|  |  |  |  |  |  |  |  | |  |  |  |  |  |  |  |
| 067 | **CD30** | 807 | 1000807 | 0.000806 | 162727.8 | 492.05777 | 0 | | 75 | 0 | 0 | 0 | 60 | 0 | 0 |
|  | **CD32** | 7910 | 1007910 | 0.007848 | 139055.6 | 4092.366 | 0 | | 75 | 0 | 0 | 0 | 60 | 0 | 0 |
|  | **CD30&CD32** | 1214 | 1001214 | 0.001213 | 171043.4 | 777.73073 | 0.197662 | | 75 | 1.48 | 1906.14 | 0.00 | 60 | 0 | 0 |
|  | **Neg** | 133409 | 1133409 | 0.117706 | 193508.7 | 85414.236 | 0.101781 | | 75 | 0.76 | 8.94 | 33.00 | 60 | 197.99 | 2318.03 |
|  |  |  |  |  |  |  |  | |  |  |  |  |  |  |  |
| 065 | **CD30** | 1669 | 1001669 | 0.001666 | 69765.4 | 435.91665 | 0 | | 75 | 0 | 0 | 0 | 60 | 0 | 0 |
|  | **CD32** | 6543 | 1006543 | 0.0065 | 92449.85 | 2253.6272 | 0.02462 | | 75 | 0.18 | 81.94 | 12.92 | 60 | 77.55 | 34410.83 |
|  | **CD30&CD32** | 1140 | 1001140 | 0.001139 | 63631.16 | 271.71346 | 0.164705 | | 75 | 1.24 | 4546.27 | 3.76 | 60 | 22.53 | 82935.08 |
|  | **Neg** | 253788 | 1253788 | 0.202417 | 138369.6 | 105031.35 | 2.43815 | | 75 | 18.29 | 174.10 | 58.16 | 60 | 348.95 | 3322.34 |
|  |  |  |  |  |  |  |  | |  |  |  |  |  |  |  |
| 066 | **CD30** | 588 | 1000588 | 0.000588 | 82154.16 | 181.04347 | 0 | | 75 | 0 | 0 | 0.00 | 60 | 0 | 0 |
|  | **CD32** | 21705 | 1021705 | 0.021244 | 98948.46 | 7882.6921 | 0 | | 75 | 0 | 0 | 4.50 | 60 | 27.03 | 3428.95 |
|  | **CD30&CD32** | 231 | 1000231 | 0.000231 | 90636.46 | 78.495697 | 0 | | 75 | 0 | 0 | 0 | 60 | 0 | 0 |
|  | **Neg** | 579489 | 1579489 | 0.366884 | 131905.7 | 181477.78 | 2.428652 | | 75 | 18.21 | 100.37 | 116.47 | 60 | 698.81 | 3850.69 |
| Input cell number - number of cells recovered from flow sorting used in extraction or spiked into uninfected carrier PBMCs (for CD30^+^ cells) to maximize DNA and RNA recovery and normalize extraction efficiency between CD30^+^ and CD30^-^ CD4^+^ T cell populations. Spiking rare cells also allowed for the input of similar amounts of RNA into each PCR reaction well.  Cell number + HIV neg spike cells= total number of cells from which HIV-1 DNA and RNA were extracted.  Cell number ratio is the ratio of sorted input cells to total cells including uninfected carrier cells (if added). This ratio is used to adjust the number of cells used in PCR analysis as determined by the CCR5 gene count.  CCR5 = conserved human DNA gene used to determine the number of cells surveyed in each PCR assay (diploid gene, so 2 DNA copies = 1 cell) in 10ul of eluted DNA.  Cell output number is the number of CD30^+^ or CD30^-^ cells surveyed per total elution volume based on spike ratio and CCR5 count.  HIV-1 DNA = mean copies per reaction well (10 µL volume) detected by rtPCR from assay replicates.  Total DNA or RNA extraction volume = total volume of eluted DNA or RNA from the AllPrep procedure.  Total DNA or RNA = the total number of DNA copies per total sample elution volume.  Total DNA or RNA per 10^6^ cells is calculated using the total DNA or RNA over the total number of cells per reaction volume. This is the final readout of the assay.  Example Calculation for PID #11 CD30^+^ Cells: Mean of 26 copies of HIV-1 DNA in 10ul input PCR assay reaction wells x 10 (to adjust for total number of copies within the 100μl of eluted DNA) which comes to 260.07 copies per 100μl of eluted DNA. Cell output number = 439.53, which is derived from total CCR5 copies (125400.1) divided by 2 (haploid gene) and multiplied by 10 given the number is based on average copies per 10μl of input DNA, from a total of 100μl total DNA elution volume. This number = 627,000.5 which are the total number of cells surveyed in the eluted DNA. This is then multiplied by the ratio of CD30^+^ to HIV uninfected carrier cells (0.000701) to make a total of 439.53 CD30^+^ cells surveyed in the eluted sample. (260.07/439.53)*1,000,000 = 591,701 HIV^+^ DNA copies per 10^6^ CD30^+^ cells. Of note, the RNA was eluted in a different volume during simultaneous extraction which is why we calculate both nucleic acid and cell copy numbers per total elution extraction volumes. | | | | | | | | | | | | | | | |
