# Supplementary material for: Increased HIV-1 transcriptional activity and infectious burden in peripheral blood and gut-associated CD4+ T cells expressing CD30
Source: PLoS Pathog. 2018 Feb 22;14(2):e1006856. doi: 10.1371/journal.ppat.1006856 (PMC5823470; doi:10.1371/journal.ppat.1006856)
Supplement: S3 Table — (DOCX) [file ppat.1006856.s003.docx]

| S3 Table: Absolute cell numbers obtained following fluorescence-activated cell sorting of rectal tissue samples. | | | | | |
| --- | --- | --- | --- | --- | --- |
| PID# | **047** | **063** | **065** | **066** | **067** |
| CD30^+^ | 899 | 4090 | 1669 | 588 | 807 |
| CD30^+^CD32^+^ | 717 | 14179 | 1140 | 231 | 1214 |
| CD32^+^ | 8510 | 12954 | 6543 | 21705 | 7910 |
| Neg | 558121 | 1229500 | 253788 | 579489 | 133409 |
