# Supplementary figures and images for: Increased HIV-1 transcriptional activity and infectious burden in peripheral blood and gut-associated CD4+ T cells expressing CD30
Source: PLoS Pathog. 2018 Feb 22;14(2):e1006856. doi: 10.1371/journal.ppat.1006856 (PMC5823470; doi:10.1371/journal.ppat.1006856)

**
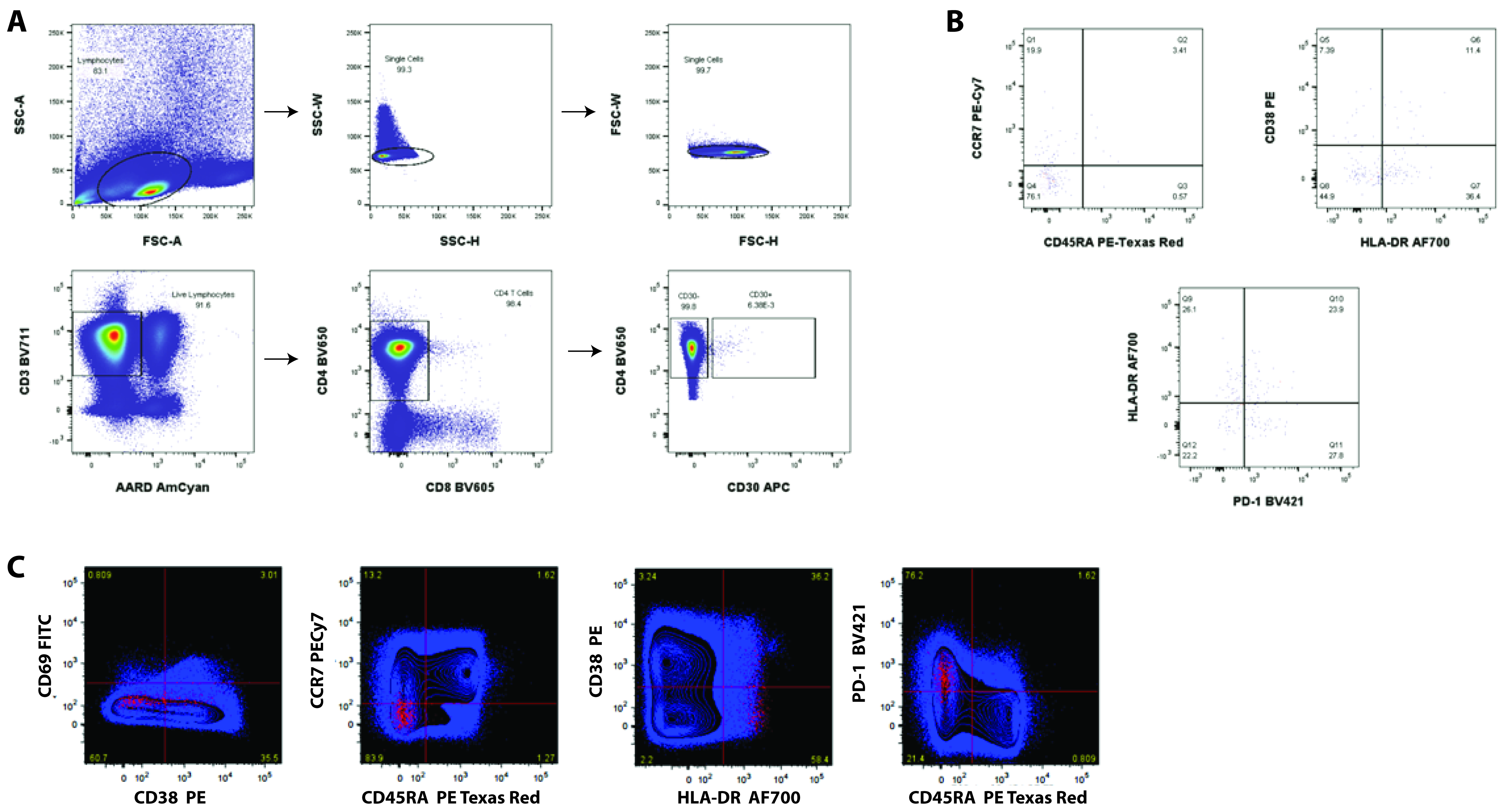
**

Supplement: S1 Fig — (A) Lymphocytes populations were first selected using forward and side scatter characteristics. Following this, the exclusion of doublets was performed by plotting cells on SSC-W versus SSC-H and similarly for FSC-W and FSC-H. Live and then CD3+ lymphocytes were selected, and further gated for CD4 expression. Following this CD4+ CD30+ T cells and CD30-CD4+ T cells were selected in individual gates and sorted (for sorting experiments). (B) For further phenotypical analysis, the same gating was applied as described in (A) and then extended, allowing the identification of CD30 expressing cells with CD45RA and CCR7 populations (T cell subsets), CD69 (early activation), CD38 and HLA-DR (Late activation) and PD-1 expression. (C) CD30 expressing cells (red) are then compared and contrasted to CD30 negative CD4+ T cell populations (Blue), shown together on the same plots. A fluorescence minus one for APC-conjugated anti-CD30 was included to establish CD30 gating. (DOCX) [file ppat.1006856.s004.docx]

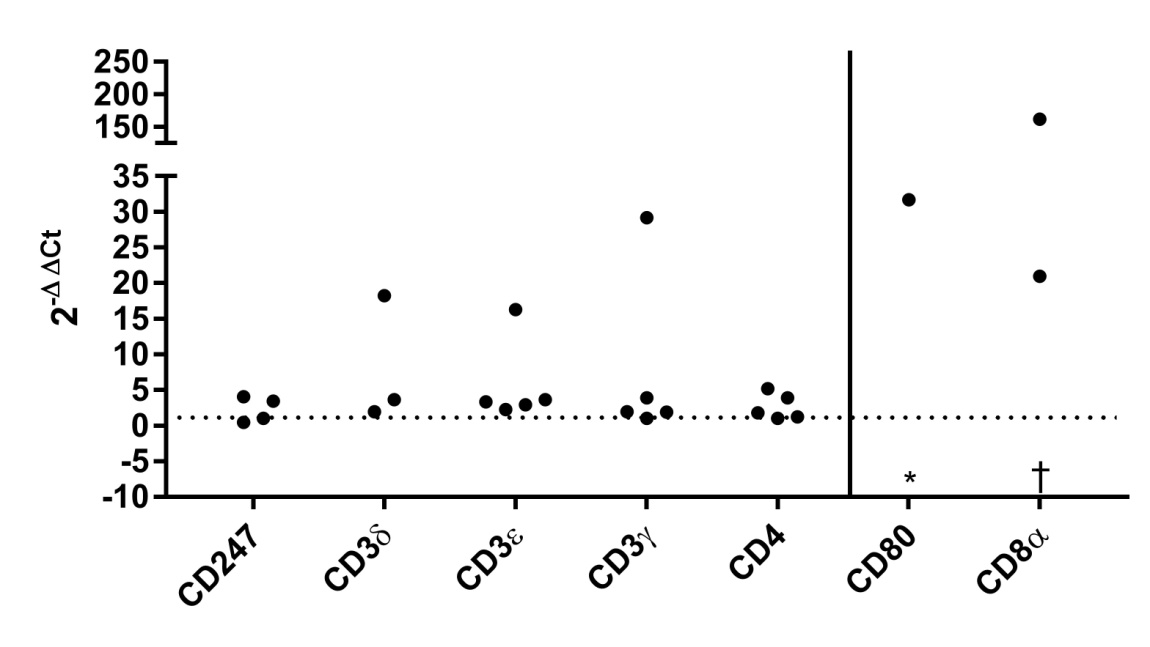

Supplement: S2 Fig — Of note, no CD80 mRNA could be detected in four samples (*) and no CD8α mRNA could be detected in three samples (†). Overall, 2-ΔΔCt values of CD3 complex and CD4 mRNA were similar or higher comparing CD30+ and CD30- CD4+ T cells. No CD3 complex, CD4 or CD8 mRNA could be detected from purified B cells obtained from an uninfected donor which served as a control. 2-ΔΔCt values represent a function comparing CD30+ to CD30- CD4+ T cell mRNA levels. A value of 1 represents no difference between populations within an individual sample and values greater than 1 indicate a greater number of mRNA transcripts. (DOCX) [file ppat.1006856.s005.docx]

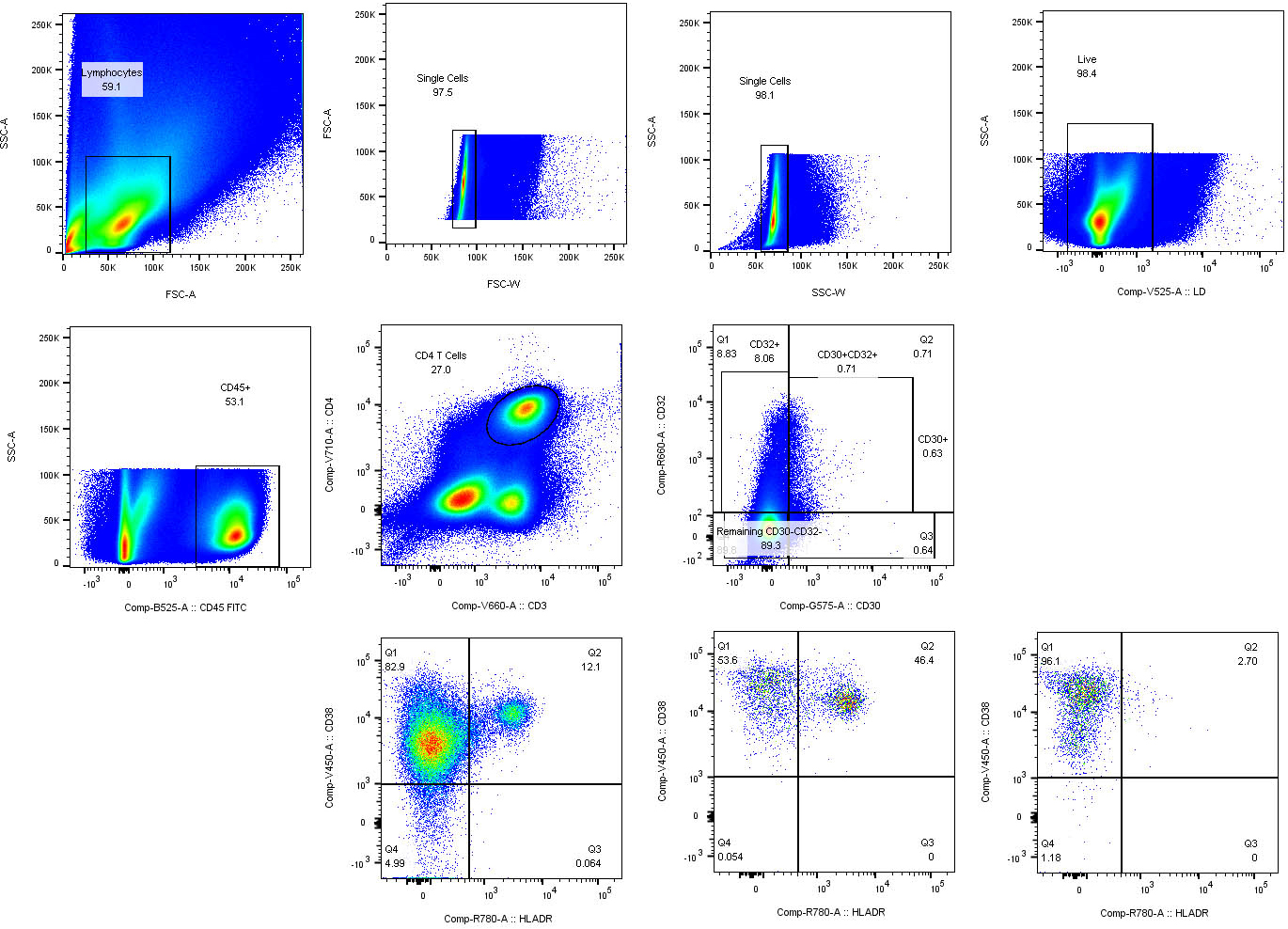

Supplement: S3 Fig — Lymphocytes populations were first selected using forward and side scatter characteristics. Following this, the exclusion of doublets was performed by plotting cells on FSC-W and FSC-A and similarly for SSC-W versus SSC-A. Live and then CD45+ lymphocytes were selected, and further gated for CD3+CD4+ T cells. Following this CD4+ CD30+, CD4+ CD32+, and CD4+ CD30+ CD32+ T cells were selected in individual gates and sorted. Further phenotypical information was collected on each T cell subset, including CD38, HLA-DR and CD13 expression. (DOCX) [file ppat.1006856.s006.docx]

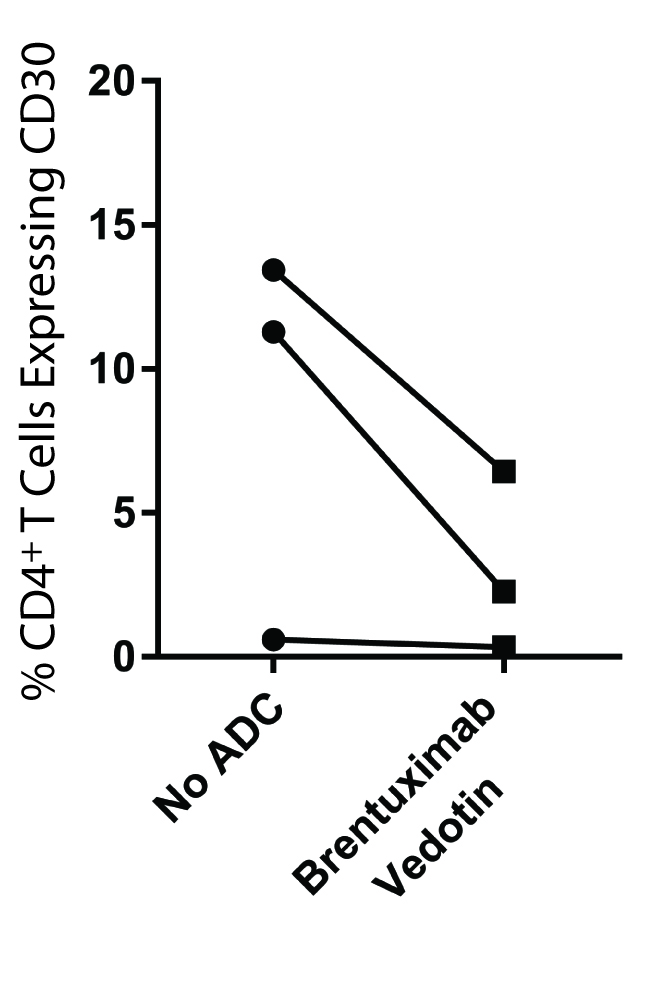

Supplement: S4 Fig — While this reduction may represent ADC-targeted cell killing, CD30 staining may also have been affected by steric interactions with ADC-bound receptor or receptor downregulation. (DOCX) [file ppat.1006856.s007.docx]
